# Single-cell atlas of reproductive endocrine organs reveals transcriptomic responses to type 1 diabetes mellitus in nonhuman primates

**DOI:** 10.1038/s41421-026-00908-2

**Published:** 2026-08-04

**Authors:** Zheng-Hui Zhao, Ning Xu, Xue-Ying Chen, Cheng-Yan Zhuo, Yong Lu, Ang Li, Qiang Sun, Xiang-Hong Ou, Qing-Yuan Sun

**Affiliations:** 1 Guangdong Provincial Engineering Research Center of Reproductive Health and Transgenerational Genetic Disease Prevention; Guangzhou Key Laboratory of Metabolic Diseases and Reproductive Health; Guangdong-Hong Kong Metabolism & Reproduction Joint Laboratory, Reproductive Medicine Center, The Affiliated Guangdong Second Provincial General Hospital of Jinan University, Guangzhou, Guangdong China; 2 Institute of Neuroscience, Center for Excellence in Brain Science and lntelligence Technology, State Key Laboratory of Neuroscience, Chinese Academy of Sciences, Shanghai, China; 3 School of Medicine/Institute of Advanced Studies Hong Kong, Sun Yat-Sen University, Hong Kong, China

**Keywords:** Mechanisms of disease, Transcriptomics

## Abstract

Type 1 diabetes mellitus characterized by insulin deficiency and hyperglycemia is associated with female subfertility. However, how hyperglycemia affects the hypothalamic-pituitary-ovarian-uterine axis remains poorly understood. In this study, we performed single-cell transcriptomic profiling of the hypothalamus, pituitary, ovary and uterus during the proliferative phase of the menstrual cycle in type 1 diabetic macaques to systematically characterize changes in tissue-specific cellular heterogeneity, gene expression, and intercellular communication networks under diabetic conditions. Our analysis revealed significant upregulation of the TNF signaling pathway across multiple tissues, concomitant with marked activation of inflammation-related pathways. Notably, the macrophage migration inhibitory factor signaling pathway exhibited a tissue-specific regulatory pattern, being significantly upregulated in the hypothalamus and pituitary but downregulated in the ovary and uterus, suggesting divergent inflammatory modulation along the reproductive endocrine axis in response to diabetes. Moreover, we observed that diabetes leads to reduced *FSHR* expression during granulosa cell differentiation, and this process is further exacerbated by the upregulated expression of *SFRP4*, a known antagonist of follicle-stimulating hormone signaling, resulting in diminished granulosa cell responsiveness to follicle-stimulating hormone. Consequently, this dysregulation is correlated with increased *FSHB* expression in pituitary gonadotropes, likely due to disrupted ovarian feedback signaling. Collectively, our findings provide a comprehensive landscape of cellular and molecular alterations in immune and endocrine compartments in the female reproductive system in diabetic states, advancing our understanding of immune‒endocrine cell crosstalk in the context of metabolic disease.

## Introduction

The female reproductive process is regulated by the coordinated actions of multiple neural and hormonal signaling pathways^1^. In the neurohormonal system, hypothalamic KISS1 neurons located in the arcuate nucleus and the anteroventral periventricular nucleus synthesize kisspeptins, which further stimulate gonadotropin-releasing hormone (GnRH) neurons to release the decapeptide GnRH, forming the central regulators of reproductive functions^2^. Subsequently, the pituitary gland, which serves as a signal mediator between the hypothalamus and the ovary, is capable of producing and releasing hormones from the anterior lobe’s endocrine cells in response to the pulsatile secretion of GnRH^3^. Next, the anterior pituitary gonadotropes secrete two gonadotropins: follicle-stimulating hormone (FSH) and luteinizing hormone (LH). These hormones promote the development of ovarian follicles during the follicular phase and the formation of the corpus luteum during the luteal phase and stimulate the production of ovarian hormones such as estradiol (E2) and progesterone (P4), respectively^4^. The follicular and luteal phases correspond to the proliferative and secretory phases of the endometrium, which is the inner mucosal lining of the uterus. In response to ovarian hormones, the functional layer of the endometrium undergoes repeated cycles of shedding, scar-free repair, and regeneration with extensive growth and differentiation^5^. Moreover, ovarian hormones also regulate the release of LH and FSH, both directly and indirectly through GnRH signaling, to dynamically modulate the function of this neurohormonal axis^6,7^. Additionally, numerous metabolic factors play crucial roles in the coordinated regulation of reproductive processes. Among these, insulin serves as a key regulator of the hypothalamic-pituitary-ovarian-uterine (HPOU) axis. It can directly influence GnRH neurons to modulate their secretory activity, thereby affecting the functions of the gonadotropic axis^8,9^.

Type 1 diabetes mellitus (T1DM) is a chronic autoimmune disease that typically develops during childhood or adolescence. T1DM is characterized by insulin deficiency and hyperglycemia, which further leads to disruptions in the reproductive endocrine system and immune responses at various levels of the HPOU axis^10–12^. Several cohort studies have shown that compared with healthy women, women with type 1 diabetes have significantly fewer offspring and a higher incidence of congenital abnormalities^13,14^. Notably, maintaining optimal metabolic control and remaining free of complications can effectively mitigate diabetes-associated subfertility among women^13^. Mechanistically, women with poorly controlled T1DM often present with hypogonadotropic hypogonadism, as evidenced by low levels of circulating LH, FSH, and estradiol^15,16^. Consistent with these clinical observations, rodent models of T1DM have revealed that female animals with uncontrolled diabetes display a profound hypogonadotropic state characterized by low basal levels of hormones^17,18^. Additionally, immune-endocrine interactions are crucial for hormone production and maintenance of endocrine homeostasis. Within the HPOU axis, macrophages are the predominant type of immune cell and play key roles in inflammatory responses and tissue homeostasis. These macrophages have varied origins and form diverse subpopulations within endocrine organs. Each adult organ contains its own unique pool of functionally distinct macrophages that control specific tissue and niche functions^19,20^. Tissue-resident macrophages typically originate from embryonic progenitor cells in the yolk sac and/or fetal liver. In contrast, monocyte-derived macrophages primarily originate from circulating monocytes that differentiate upon migrating into various organs^21^. Tissue-resident macrophages are maintained in tissues through local self-proliferation or monocyte recruitment from blood circulation^22^. However, monocytes are unable to enter the hypothalamus and pituitary; therefore, the macrophages in this unique immune environment are maintained primarily through self-proliferation^23–25^. In contrast, the macrophages in ovaries maintain themselves through both the self-proliferation of tissue-resident macrophages and the differentiation of circulating monocytes^26^. Although several research groups have provided detailed cell atlases of organs within the HPOU axis^26–29^, the changes in reproductive endocrine functions and the inflammatory characteristics of the immune microenvironment in T1DM primates are still not known. This highlights the need for investigations into the underlying mechanisms through which T1DM affects immune-endocrine interactions in primates.

Therefore, we utilized single-cell RNA sequencing (scRNA-seq) to systematically profile hypothalamus, pituitary, ovary, and uterus tissues and dissect T1DM-related changes in cellular composition and the immune-endocrine interaction mechanisms involved in regulating reproductive processes. We identified a substantial number of differentially expressed genes (DEGs) and biological pathways, providing a comprehensive view of the differences between control subjects and those with T1DM. Leveraging these high-resolution datasets, we found that diabetes significantly impairs the responsiveness to FSH. Correspondingly, the disruption of follicular development is accompanied by increased *FSHB* expression in pituitary gonadotropes, highlighting systemic reproductive impairments in diabetic individuals. Our findings clarify how T1DM affects reproductive health at the cellular and molecular levels, paving the way for more targeted therapeutic strategies.

## Results

### Transcriptome profiling of diabetic reproductive endocrine organs

**Fig. 1:**
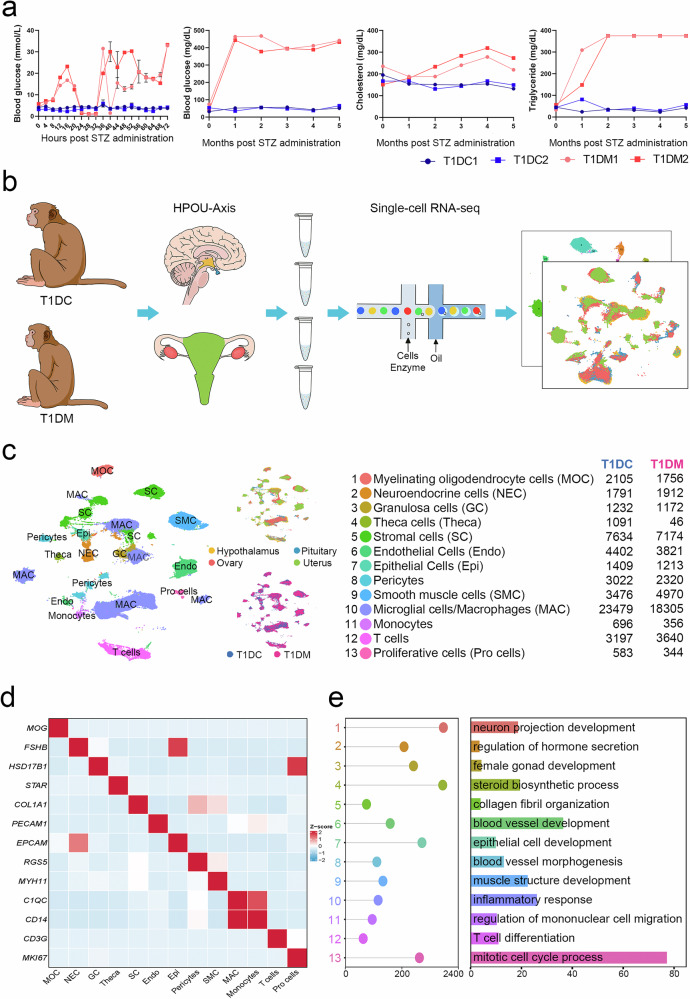
Single-cell transcriptomic atlas of the HPOU axis in diabetic macaques. **a** Dynamic alterations in fasting blood glucose, cholesterol, and triglyceride levels in nonhuman primate (NHP) with T1DM. **b** Schematic diagram of the experimental design and workflow for scRNA-seq. **c** Unsupervised clustering analysis of 101,146 cells (divided into 13 clusters) from pooled samples of the HPOU axis, colored by cell type. The results in the middle show the distribution of cells in each organ and group. **d** Heatmap of the expression of cell type-specific marker genes. **e** Lollipop chart of the number of cell marker genes (left panel) and bar plot of the cell type-specific enriched biological processes.

We utilized four female cynomolgus monkeys aged 5–6 years in our study, including two controls and two streptozotocin (STZ)-induced type 1 diabetic macaques. For the cynomolgus monkeys in the T1DM group, intravenous administration of STZ (80 mg/kg/body weight) was performed to induce T1DM. Compared with T1DM control (T1DC) monkeys, diabetic monkeys presented significantly increased fasting blood glucose, triglyceride, and cholesterol levels (Fig. 1a; Supplementary Fig. S1a). C-peptide levels and body weights were significantly lower in diabetic monkeys than in T1DC monkeys (Supplementary Fig. S1a). Additionally, while the levels of blood urea nitrogen (BUN) remained unchanged, the liver enzymes alanine aminotransferase (ALT) and aspartate aminotransferase (AST) were slightly elevated in the T1DM group (Supplementary Fig. S1b). Moreover, compared with normal physiological ranges, menstrual cycle disorders, manifested as either prolonged or shortened cycle lengths, were also observed in the T1DM group (Supplementary Fig. S1c). Next, we used scRNA-seq (10X Genomics Chromium system) to profile single-cell suspensions from hypothalamic, pituitary, ovarian and uterine tissues (Fig. 1b; Supplementary Table S1). After quality control, doublet removal and ambient RNA decontamination (Supplementary Fig. S1d), a total of 101,146 cells were retained for downstream analysis. We integrated these cells into a SCTransform-normalized dataset and further subjected them to principal component analysis (PCA) for dimensional reduction, and 13 distinct clusters were generated using unsupervised graph-based clustering (Fig. 1c). Visualization of the uniform manifold approximation and projection (UMAP) plot illustrated the distribution of different cell types, tissues, and experimental groups (Fig. 1c). Cluster analysis enabled the annotation of major cell types on the basis of the expression of canonical markers, including myelinating oligodendrocyte cells (MOCs; *n* = 3861; *MOG*^+^), neuroendocrine cells (NECs; *n* = 3703; *FSHB*^+^), granulosa cells (GCs; *n* = 2404; *HSD17B1*^+^), theca cells (theca; *n* = 1137; *STAR*^+^), stromal cells (SCs; *n* = 14,808; *COL1A1*^+^), endothelial cells (Endo; *n* = 8223; *PECAM1*^+^), epithelial cells (Epi; *n* = 2622; *EPCAM*^+^), pericytes (*n* = 5342; *RGS5*^+^), smooth muscle cells (SMCs; *n* = 8446; *MYH11*^+^), microglia and macrophages (MACs; *n* = 41,784; *C1QC*^+^), monocytes (*n* = 1052; *CD14*^+^), T cells (*n* = 6837; *CD3G*^+^) and proliferative cells (Pro cells; *n* = 927; *MKI67*^+^) (Fig. 1d; Supplementary Fig. S1e and Table S2). Furthermore, we identified a list of significantly DEGs for each cell type (*P*_val < 0.01 and avg_log_2_fold change (FC) ≥ 0.25), followed by Gene Ontology (GO) enrichment analysis to characterize the associated biological functions. The enriched cellular and biological processes were highly consistent with the cell type identities, such as the regulation of hormone secretion in neuroendocrine cells and epithelial cell development in epithelial cells (Fig. 1e).

### Transcriptomic susceptibility of different cell types to diabetes

**Fig. 2:**
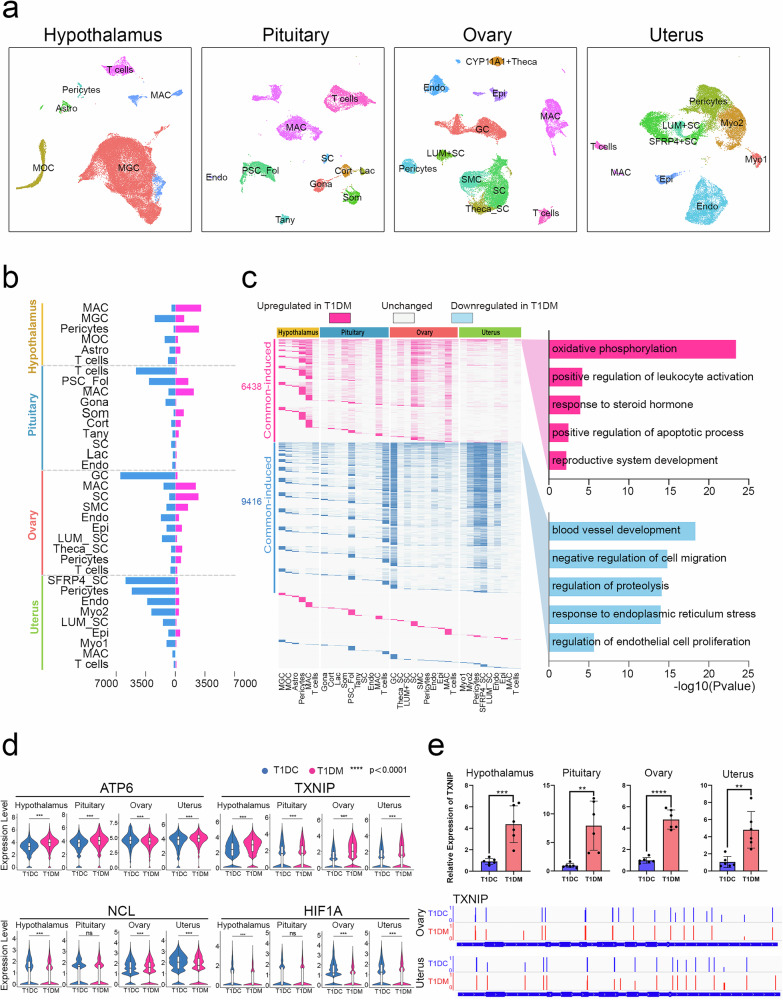
Diabetes-associated transcriptional alterations in various cell types. **a** UMAP plots showing the cellular heterogeneity of each reproductive endocrine organ, with cells color-coded according to the main cell types identified. **b** Number of upregulated (red bar) and downregulated (blue bar) genes in each cell type. **c** Distribution of DEGs in a heatmap indicating whether a gene (row) is a DEG in a given cluster (column) (left). GO terms associated with the top 800 commonly induced DEGs (right). **d** Violin plots showing upregulated and downregulated DEGs on the HPOU axis. **e** Expression levels of *Txnip* genes in the HPOU axis and DNA methylation profiles of *Txnip* genes in the ovary and uterus.

To elucidate the cellular and molecular changes associated with diabetes, we first investigated the diverse cellular landscape of reproductive endocrine organs. Unbiased clustering revealed tissue-specific cell types (Fig. 2a; Supplementary Fig. S2a), and the numbers of immune cells in the hypothalamus, pituitary and ovary and pericytes in the uterus significantly decreased (Supplementary Fig. S2b). Next, we sought to characterize diabetes-associated transcriptional alterations in individual organs within the HPOU axis. We found that uterine tissues presented the greatest number of DEGs, and the majority of the DEGs were downregulated (Supplementary Fig. S2c). To elucidate the transcriptional responses of the HPOU axis to diabetes, we identified DEGs in each reproductive endocrine organ (Supplementary Fig. S2d). Global analysis of DEGs and GO enrichment patterns revealed that upregulated genes overlapping across two or more tissues were predominantly enriched in pathways related to “oxidative phosphorylation” and “regulation of leukocyte activation”, whereas commonly downregulated DEGs were enriched in “blood vessel development” and “activation of immune response” (Supplementary Fig. S2d and Table S3).

Heterogeneous cell populations within the HPOU axis have been previously associated with distinct regulatory roles in reproductive endocrine function. To further dissect the transcriptional alterations associated with diabetes, we conducted cell type-specific differential expression analysis between T1DM and T1DC macaques (Fig. 2b). The cell types with a greater number of DEGs during diabetes included microglial cells (MGCs) and macrophages (MACs) in the hypothalamus; pituitary stem cells and folliculostellate cells (PSC_Fol), MACs and gonadotropes (Gona) in the pituitary; granulosa cells (GCs), MACs, smooth muscle cells (SMCs), and stromal cells (SCs) in the ovary; and *SFRP4*^+^ stromal cells (SFRP4_SCs), pericytes, endothelial cells (Endo) and myometrial cell2 (myo2) in the uterus (Fig. 2b). Furthermore, we identified diabetes-associated transcriptional changes across multiple cell types, and compared with the T1DC group, most DEGs were commonly induced (differentially expressed in at least one type) genes (Fig. 2c). Moreover, we performed GO analysis on the top 800 commonly induced genes and found that the upregulated DEGs are involved in “oxidative phosphorylation” and “response to steroid hormone”, which can be used as hallmarks of the HPOU axis in diabetes (Fig. 2c; Supplementary Table S3). Meanwhile, example genes associated with GO terms are listed, including *TXNIP*, *ATP6*, *NCL* and *HIF1A*, which represent the genes whose expression is induced by diabetes (Fig. 2d). We also observed significantly elevated *Txnip* expression in the hypothalamus, pituitary, ovary, and uterus tissues of type 1 diabetic mice via qPCR (Fig. 2e). Considering the disruption of DNA methylation in diabetic macaques, we next performed whole-genome bisulfite sequencing (WGBS) on ovarian and uterine tissues. The distribution of differentially methylated regions (DMRs) was examined, and the DNA methylation levels in the whole genome, promoter and exons significantly decreased in the ovary but did not significantly decrease in the uterus (Supplementary Fig. S2e, f). Moreover, the genomic distribution profile of DNA methylation signals at the *TXNIP* locus revealed a distinct pattern between the groups. As shown in the track view, the DNA methylation signal intensity in diabetic ovaries appeared lower than that in control ovaries (Fig. 2e). Taken together, these transcriptional responses suggest the heterogeneity of reproductive endocrine organs in response to diseases.

### Global alterations in the intercellular signaling network within the reproductive endocrine axis

**Fig. 3:**
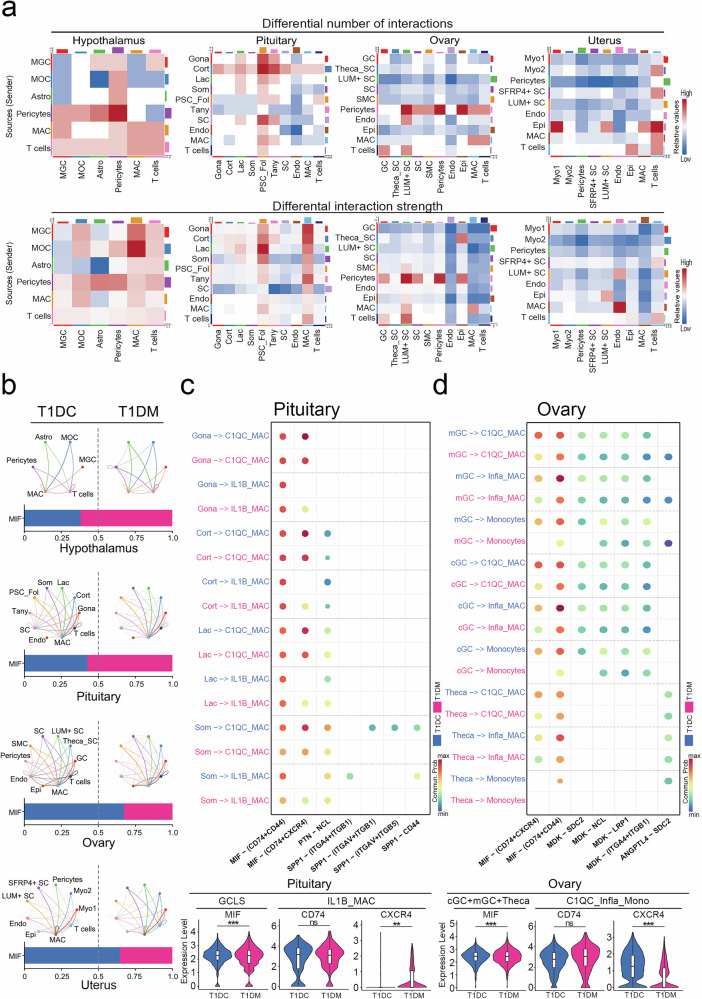
Global cell–cell interactions in the diabetic HPOU axis. **a** Heatmaps showing the differential interaction numbers and strength between the T1DM and T1DC groups. **b** Representative information flow for MIF signaling pathways in T1DC (blue) and T1DM (red) groups. **c** Ligand–receptor signaling pairs between endocrine cells and macrophages in the pituitary (top); changes in the MIF signaling pathway in the pituitary between the diabetic and control groups (bottom). **d** Ligand–receptor signaling pairs between endocrine cells and macrophages as well as monocytes in the ovary (top); changes in the MIF signaling pathway in the ovary between the diabetic and control groups (bottom).

The intricate network of interactions among various cell types within the HPOU axis is essential for maintaining normal reproductive function. However, T1DM may disrupt these interactions, and understanding how these interactions lead to dysregulation of reproductive function is currently an area of significant interest. Therefore, we performed a CellChat analysis to construct ligand–receptor interaction networks and identify key signaling pathways that were significantly altered in T1DM monkeys compared with T1DC monkeys. Our analysis revealed a marked decrease in the overall number and strength of intercellular interactions in the ovaries and uterus from the T1DM group, and increased numbers and strength of communication were observed in the diabetic hypothalamus, suggesting a compartment-specific disruption of neuroendocrine-reproductive networks under diabetic conditions (Supplementary Fig. S3a). Of particular interest, compared with those in the T1DC group, pericytes in the hypothalamus and uterus, PSC_Fol in the pituitary, and Endo in the pituitary, ovary and uterus, exhibited significant change in the number of interactions and the increased strength of communication with other cell types in the T1DM group (Fig. 3a). Subsequent analysis of differentially activated signaling pathways revealed a marked upregulation of the TNF signaling cascade in the T1DM group, suggesting a potential imbalance in inflammatory signaling pathways (Supplementary Fig. S3b). Furthermore, our results revealed that migration inhibitory factor (MIF) signaling was upregulated in the diabetic hypothalamus and pituitary, whereas it was downregulated in the diabetic ovary and uterus (Fig. 3b).

Given the critical role of interactions between immune cells and endocrine cells in maintaining hormonal homeostasis, we next sought to dissect the specific interactions between hormone-producing cells and macrophages, with a focus on Gona, corticotropes (Cort), lactotropes (Lac) and somatotropes (Som) in the pituitary and theca cells and granulosa cells in the ovary (Supplementary Fig. S3c). To characterize the distinct modes of immunomodulatory crosstalk within the pituitary microenvironment, we first investigated the differential intercellular communication patterns between Gona (senders) and Lac (senders) and MACs (receivers) (Fig. 3c). Our analysis revealed that Gona interacts mainly with MACs through the MIF signaling pathway and that Lac cells not only utilize the MIF pathway but also preferentially engage with MACs via the PTN-NCL pathway. Notably, several pituitary endocrine cell types, including Gona, Cort, Lac and Som, exhibited significantly upregulated crosstalk with IL1B_MAC through the MIF signaling pathway in the T1DM group (Fig. 3c). To elucidate the molecular mechanisms underlying this disruption, we systematically assessed the expression profiles of both MIF and its canonical receptor complex components in the relevant cellular compartments. We found that *CXCR4* expression was significantly increased in IL1B_MAC cells under diabetic conditions, suggesting that the upregulation of MIF signaling in the diabetic pituitary is predominantly driven by the increased expression of the receptor at the receiver cell level (Fig. 3c). We then extended our analysis to examine the intercellular communication between ovarian endocrine cells and ovarian MACs and monocytes. Our results indicated that these cells predominantly interacted with immune cell populations through the midkine (MK) and MIF pathways (Fig. 3d). Interestingly, we observed a substantial loss of MIF signaling between both granulosa and theca cells (sender) and several MAC subpopulations (receiver) in the T1DM group (Fig. 3d). To further dissect the molecular basis of this impairment in signaling, we systematically assessed the expression levels of both MIF and its receptor components in the corresponding cellular compartments. We found that the expression of its key receptor subunit, *CXCR4*, was notably reduced in both MACs and monocytes (C1QC_Infla_Mono) (Fig. 3d). These findings suggest that the impairment of MIF signaling in diabetic ovaries is attributable mainly to decreased receptor availability at the target cell level. On the other hand, we also explored the differential intercellular communication patterns between MACs (senders) and endocrine cells (receivers) in the pituitary and ovary. We observed that NAMPT-INSR signaling between IL1B_MAC (senders) and Cort (receivers) was significantly downregulated, whereas POMC-OPRL1 signaling was significantly upregulated in the pituitary. These changes were likely attributable to the decreased expression of NAMPT and the increased expression of POMC in IL1B_MAC cells (Supplementary Fig. S3d). In the ovary, EREG-EGFR signaling between MAC/Mono (senders) and theca and granulosa cells (receivers) was significantly upregulated in the T1DM group, which was likely driven by the markedly increased expression of EREG in immune cells (C1QC_Infla_Mono) (Supplementary Fig. S3e).

### Structural and molecular alterations in reproductive-related cells under diabetic conditions

**Fig. 4:**
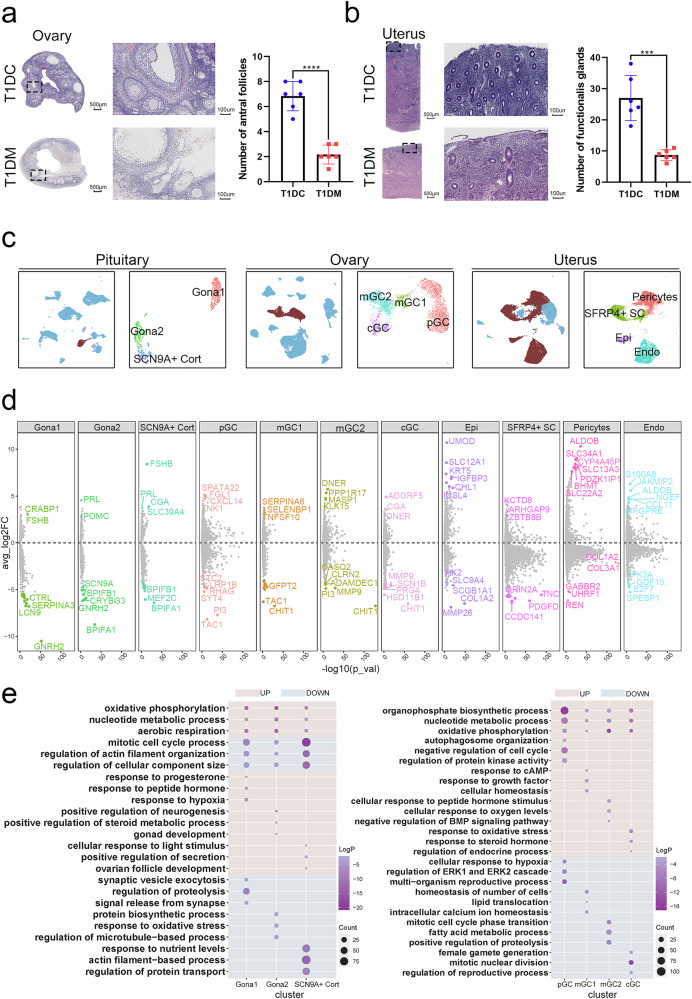
Transcriptional alterations in reproductive-related cells. **a** Left: H&E staining of the ovaries in the control and diabetic groups; right: corresponding statistics of ovarian antral follicles in the control and diabetic groups. ****P < 0.0001. **b** Left: H&E staining of the uterus in the control and diabetic groups; right: corresponding statistics of uterine functionalis glands in the control and diabetic groups. ***P < 0.001. **c** Highlight of the cells in the pituitary, ovary and uterus from Fig. 2a, showing the cells selected for focused analysis in the left panel; UMAP visualization of subtypes of these cell populations in the right panel. **d** Volcano plots showing the DEGs of T1DM vs T1DC in each subtype. Representative upregulated and downregulated genes are labeled. **e** GO terms associated with DEGs in the indicated pituitary and ovarian clusters.

To further assess the impact of T1DM on reproductive function, we performed ultrasound imaging alongside histopathological analysis of hypothalamic, ovarian and uterine sections using hematoxylin and eosin (H&E) staining. The T1DM group exhibited pronounced morphological alterations, characterized by ovarian and uterine atrophy (Supplementary Table S4), a significant loss of ovarian antral follicles, and a reduction in uterine functionalis glands, reflecting severe structural impairments induced by diabetes (Fig. 4a, b). To elucidate the reproductive-related cell-specific molecular characteristics of the T1DM group, we isolated Gona from the pituitary and granulosa cells from the ovary, and subsequently categorized them into seven subtypes (Fig. 4c). Given that the endometrium undergoes cyclic changes in response to ovarian hormones, we focused on investigating alterations in epithelial cells, stromal cells, and vascular-related cells in the uterus in the T1DM group (Fig. 4c).

To systematically compare the transcriptional responses across distinct reproductive-related cell populations under T1DM and T1DC conditions, we performed DEG analysis of the T1DM vs T1DC groups among different cell clusters separately and generated an integrated volcano plot encompassing eleven key cell types from the pituitary, ovary, and uterus (Fig. 4d). We found that the expression of hormone-related genes, such as *FSHB* in Gona1 and *SCN9A*^+^ Cort, was upregulated in the T1DM group. Consistent with these findings, serum FSH levels were higher in the T1DM group than in the control group (Supplementary Fig. S4a). Notably, the expression of *BPIFA1*, a secreted biomarker, was downregulated in both Gona2 and *SCN9A*^+^ Cort (Fig. 4d), which may be correlated with the reduction in the number of secretory cells and anti-inflammatory functions^30,31^. In ovarian subtypes, *CXCL14 is* upregulated in preantral follicle granulosa cells (pGC), whereas *CHIT1* is downregulated in mural granulosa cells 1 and 2 (mGC1 and mGC2) and cumulus granulosa cells (cGC). Moreover, the DEG analysis of the uterus revealed that the expression of collagen family-related genes, such as *COL1A2* and *COL3A1*, was downregulated in uterine Epi cells and pericytes (Fig. 4d).

The diverse transcriptional responses to diabetes in these reproductive-related cells provide a valuable resource for identifying candidate regulators and pathways that may drive cell-specific dysfunction in the diabetic reproductive system. We subsequently performed an overlap analysis between the identified DEGs and a transcription factor database derived from macaques, revealing that the majority of the differentially expressed transcription factors were downregulated in the T1DM group (Supplementary Fig. S4b). Furthermore, we performed GO analysis across these subtypes and revealed that the “response to progesterone” and “response to peptide hormone” in Gona1 cells, “ovarian follicle development” in *SCN9A*^+^ Cort, “response to steroid hormone” in cGC, “response to peptide hormone” in *SFRP4*^+^ SC, and “sterol metabolic process” in pericytes were markedly upregulated (Fig. 4e; Supplementary Fig. S4c). These results suggest that T1DM induces widespread alterations in steroid and peptide hormone signaling pathways in a cell-specific manner, potentially contributing to impaired endocrine regulation in the reproductive endocrine axis.

### Immune microenvironment disorders associated with diabetes

**Fig. 5:**
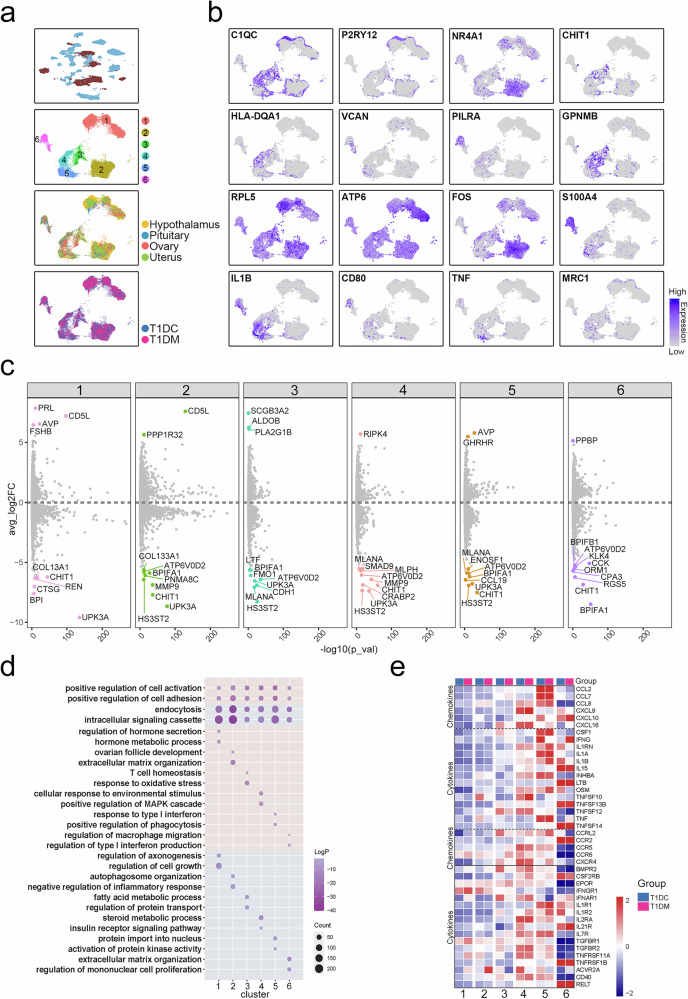
Diversity of macrophages at the molecular and functional levels. **a** Highlight of macrophages in the UMAP from Fig. 1c, showing the cells selected for focused analysis in the top panel; UMAP visualization of macrophage derivation and subtypes in the middle panels; UMAP visualization of the group of macrophages in the bottom panel. **b** Expression of selected markers identifying macrophage subtypes projected on the UMAP plot. Purple (or gray) represents a high (or low) expression level, as shown on the color key at the bottom right. **c** Volcano plots showing the DEGs of T1DM vs T1DC for each macrophage subtype. **d** GO terms associated with DEGs in the indicated macrophage clusters. **e** Heatmap showing the differentially expressed chemokines, cytokines, and their receptors in different macrophage subtypes.

The immune microenvironment plays a crucial role in the regulation of homeostasis within the reproductive endocrine system. To gain a deeper understanding of the molecular changes in macrophages between T1DC and T1DM samples, we isolated MACs and categorized them into six subtypes on the basis of marker genes and associated GO terms (Fig. 5a, b; Supplementary Fig. S5a). To further understand the alterations in different macrophage subtypes between the T1DC and T1DM groups, we performed correlation analysis. We observed that in the T1DM group, cluster 3 (C3), C4 and C5 exhibited weaker correlations with C6 (Supplementary Fig. S5b). After annotation, we found that the relative proportions and actual numbers of macrophage subtypes significantly differed between T1DC and T1DM monkeys. We observed a significant reduction in the number of C2 in the hypothalamus, C6 in the pituitary, C4 in the ovary and C5 in the uterus and a significant increase in the number of C1 in the hypothalamus and pituitary, C6 in the ovary and C2 in the uterus in the T1DM group (Supplementary Fig. S5c). Next, we performed DEG analysis of the T1DM vs T1DC groups among different MAC subclusters separately (Fig. 5c). We detected the downregulation of immune-associated genes such as *BPIFA1* and *CHIT1* in the majority of MAC subtypes. In contrast, we also observed increased expression levels of *PRL* and *FSHB* in C1 and *GHRHR* in C5, suggesting that these two macrophage subpopulations may be closely involved in endocrine regulation (Fig. 5c). Furthermore, GO analysis of the MAC subtypes revealed that upregulated DEGs were enriched in the “positive regulation of cell activation” category, whereas downregulated DEGs were enriched in the “intracellular signaling cassette” category. Specifically, “regulation of hormone secretion” in C1 and “ovarian follicle development” in C2 were upregulated, whereas “negative regulation of inflammatory response” in C2 and “fatty acid metabolic process” in C3 and “steroid metabolic process” in C4 were downregulated (Fig. 5d). To further explore the effect of T1DM on the different MAC subtypes, we evaluated the effect of T1DM on the chemokine and cytokine interactions between immune cells (Fig. 5e). We observed that alterations in cytokines and chemokines predominantly occurred in the C4, C5, and C6 subpopulations. Notably, C4 and C5 exhibited similar trends, whereas C6 displayed the opposite pattern. For instance, the expression of *IL1B*, *OSM*, *CCRL2*, *CXCR4*, *IL1R1*, and *IL1R2* was upregulated in the C5 subpopulation of the T1DM group but downregulated in the C6 subpopulation (Supplementary Fig. S5d). Conversely, the expression of *CXCL10*, *IFNG*, and *ACVR2A* was downregulated in the C5 T1DM group but upregulated in the C6 T1DM group (Fig. 5e). These findings reveal a distinct and opposing dysregulation of inflammatory mediators between the C5 and C6 subpopulations in T1DM monkeys, suggesting their potentially divergent roles in disease immunopathogenesis.

### Reduced FSH responsiveness in granulosa cells under diabetic conditions

**Fig. 6:**
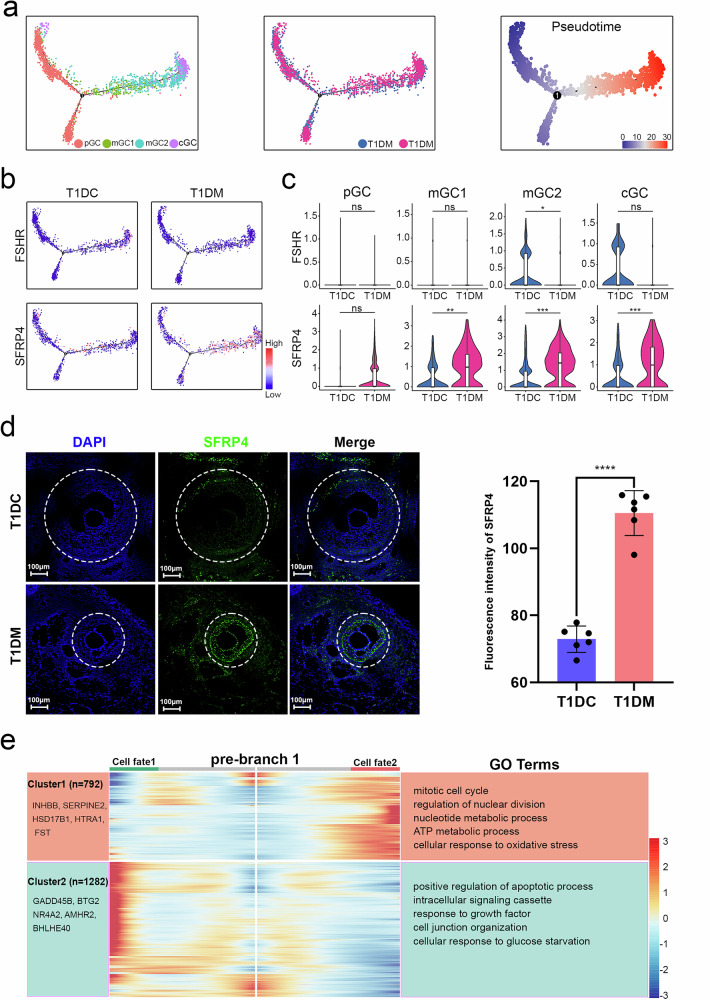
Molecular changes in granulosa cells in diabetes. **a** Pseudotime trajectory of granulosa cells analyzed by Monocle. **b** Expression of hormone-responsive genes along a pseudotime trajectory. **c** Violin plots of the expression of hormone-responsive genes between the T1DC and T1DM groups. **d** Immunostaining of SFRP4 in ovary sections (left). The DNA was counterstained with DAPI. Scale bars, 100 µm. The mean fluorescence intensity of SFRP4 (right) was calculated by ImageJ (*n* = 6). ****P < 0.0001. **e** Pseudotemporal expression pattern analysis of the representative DEGs in 2 clusters, along with the inferred trajectory and enriched biological process terms.

Given the observed follicular developmental abnormalities in diabetic ovaries, we next sought to dissect the cellular and molecular alterations underlying granulosa cell differentiation in the T1DM group. To further characterize the impact of diabetes on granulosa cell differentiation, we utilized the re-clustered granulosa cells shown in Fig. 4c and constructed a pseudotime trajectory using Monocle 2 in both the T1DC and T1DM groups (Fig. 6a). Striking differences were observed in the inferred trajectories of granulosa cells, and the diabetic granulosa cells exhibited an abnormal maturation process. Specifically, the expression of *FSHR*, a central mediator of FSH signaling, was significantly downregulated in mGC2 from the diabetic group, whereas the expression of *SFRP4*, an extracellular inhibitor of WNT signaling implicated in antagonizing FSH activity^32^, was markedly upregulated in mGC1, mGC2 and cGC (Fig. 6b, c). Consistent with these findings, immunofluorescence staining showed a marked increase in SFRP4 protein levels in ovarian follicles from the T1DM group, corroborating our transcriptomic findings (Fig. 6d). Furthermore, downregulation of *INHBB* expression was observed specifically in cGC from the T1DM group, suggesting impaired differentiation of granulosa cells (Supplementary Fig. S6a, b). Additionally, the upregulation of *AMHR2* in diabetic pGC may represent a compensatory mechanism to counteract aberrant follicular hyperactivation and preserve follicular homeostasis under metabolic stress conditions (Supplementary Fig. S6a, b). Interestingly, the expression of *HSD17B1* increased in mGC1 in the T1DM group; in contrast, a significant reduction in expression was observed in cGC (Supplementary Fig. S6a, b). To further characterize the dynamic transcriptional landscape of granulosa cell differentiation, we conducted pseudotime-dependent differential expression analysis and GO enrichment profiling across trajectory clusters (Fig. 6e). C1 consisted of genes such as *INHBB*, *HSD17B1*, and *FST*, which were highly expressed at the terminal stage and enriched in biological processes related to “mitotic cell cycle” and “cellular response to oxidative stress”. C2 contained genes such as *AMHR2* and *GADD45B*, which were predominantly expressed at the early stages of the trajectory and are known to be involved in “response to growth factor” and “cell junction organization” biological processes (Fig. 6e). Collectively, these findings reveal a profound effect of diabetes on the hormonal regulatory landscape of ovarian granulosa cells.

## Discussion

The utilization of scRNA-seq technology has revolutionized our ability to dissect the molecular identity and functional states of diverse cell populations at high resolution. In this study, we systematically investigated the cellular and molecular changes associated with their vulnerability to diabetic perturbations across the HPOU axis. Overall, we revealed critical abnormalities, including in hormone synthesis, secretion and responsiveness (Fig. 4d, e), the immune microenvironment and granulosa cell differentiation (Figs. 5, 6a, b). Moreover, we also observed persistent upregulation of “oxidative phosphorylation” in the majority of cell types from the diabetic group (Fig. 2c). These alterations may further induce reproductive endocrine dysfunction.

Reproductive impairment in T1DM is primarily attributed to insulin deficiency and metabolic disturbances driven by hyperglycemia. Given that uncontrolled T1DM is strongly associated with the development of hypogonadotropic hypogonadism^33^, stricter metabolic control emerges as a critical strategy to prevent subsequent subfertility^13^. Since insulin receptors are ubiquitous in the hypothalamus, pituitary, ovary, and uterus, the absence of endogenous insulin could impair reproductive function by depriving the HPOU axis of essential signaling^34^. For example, insulin binds to IGF1 receptors in the ovary and acts as a co-gonadotropin to stimulate androgen secretion and increase the activity of several steroidogenic enzymes^35,36^. Additionally, hyperglycemia may induce glucotoxicity in GnRH neurons, leading to a decreased LH response to GnRH stimuli and further resulting in amenorrhea^37^. Notably, the observation that the pituitary gland in individuals with T1DM retains normal responsiveness to exogenous GnRH supports the hypothesis that the associated hypogonadism originates from hypothalamic dysfunction rather than pituitary failure^38^.

Nonhuman primate (NHP) models of diabetes mellitus are indispensable for advancing our understanding of disease pathogenesis and for evaluating the efficacy and safety of novel therapeutic strategies. NHP models of type 1 diabetes are primarily established by administering STZ to destroy pancreatic β-cells; however, the dosage of STZ varies depending on factors such as species, body weight, and age^39,40^. In addition to hyperglycemia, T1DM is also associated with metabolic abnormalities such as hyperlipidemia and hypertriglyceridemia^41,42^, which is consistent with our findings. Furthermore, our data revealed significant activation of the TNF signaling pathway in diabetic monkeys, indicating that chronic inflammation is a prominent feature of the diabetic milieu. This observation is corroborated by the findings of Lei et al.^27^, whose spatial transcriptomic analysis demonstrated microglial activation and neuronal inflammatory responses in the hypothalamus of diabetic macaques.

The immune microenvironment is not only essential for the development and normal function of endocrine tissues under physiological conditions, but also plays pivotal roles in the pathogenesis of various endocrine-related disorders. As key components of the innate immune system, macrophages play diverse and critical roles in maintaining homeostasis throughout the body. In particular, they are known to reside within endocrine glands, where emerging evidence highlights their close interactions with endocrine cells^43^. Notably, macrophages contribute significantly to the regulation of the reproductive endocrine system, influencing hormone production, tissue remodeling, and local immune tolerance. In this study, we dissected the cellular heterogeneity and molecular signatures of several MAC subpopulations to understand the changes in the immune microenvironment across the HPOU axis under diabetic conditions. We observed that the diabetic condition was associated with a marked decrease in the cellular abundance of hypothalamic microglia and pituitary and ovarian macrophages within the reproductive endocrine axis (Supplementary Figs. S2b, S5c). Moreover, the TNF signaling pathway was significantly upregulated in the hypothalamus, ovary and uterus of the T1DM group (Supplementary Fig. S3b). TNF-α, produced mainly by macrophages, is a pro-inflammatory cytokine, and TNF-α inhibitors have demonstrated efficacy in clinical trials for T1DM^44,45^. Moreover, TNF-α-knockout mice exhibited increased proliferation of granulosa cells and decreased apoptosis of oocytes, which further increased the fertility of these mice^46^. Furthermore, we also examined hormone-producing and secretory cell populations in the pituitary and ovary, including gonadotropes and granulosa cells. Notably, GO enrichment analysis revealed that upregulated DEGs in these cells were predominantly associated with hormone-responsive pathways, suggesting a potential adaptive or compensatory mechanism in the HPOU axis under diabetic conditions (Fig. 4e). These findings provide novel insights into the cellular and molecular alterations associated with T1DM and highlight the importance of investigating how interactions between hormonal and immune networks are altered in T1DM.

The ovary plays a central role in the synthesis and regulation of steroid hormones, including E2 and P4, which are critical for follicular development, endometrial proliferation, and the neuroendocrine feedback that governs the HPOU axis. In the ovary, granulosa cells represent a key cellular hub for hormone production and response, with FSH signaling via FSHR being essential for aromatase activation, estrogen biosynthesis, and follicular maturation. In this study, a key finding is the marked downregulation of the expression of *FSHR*, the primary receptor for FSH, in mGC2 cells from the T1DM group (Fig. 6b). Given that FSH signaling via the FSHR is indispensable for granulosa cell proliferation, differentiation, and steroidogenesis, this reduction in receptor expression likely contributes to impaired responsiveness to gonadotropic stimulation and disrupted follicular development under diabetic conditions. Importantly, this decrease in *FSHR* expression was accompanied by the significant upregulation of the expression of *SFRP4* (Fig. 6b, c), an extracellular antagonist of WNT signaling that has been previously shown to antagonize the activity of FSH through the GSK3β-AMPK-AKT signaling pathway^32^. Collectively, these molecular alterations, such as reduced *FSHR* and elevated *SFRP4*, indicate a state of diminished hormonal sensitivity in granulosa cells from diabetic ovaries and suggest that targeting *SFRP4* or restoring WNT/FSH crosstalk could represent novel therapeutic strategies for improving follicular health in metabolic disorders.

To conclude, our profiling of single-cell atlases reveals the molecular changes in the HPOU axis in diabetic monkeys and provides potential candidate therapeutic targets or biomarkers for the evaluation of diabetic effects. Our study also sheds light on how macrophages may impact HPOU axis functionality at single-cell resolution, which may be helpful for better understanding the effects of diabetes on the functions of the reproductive endocrine system. Notably, any window of exposure to T1DM may, to some extent, lead to decreased fertility; therefore, it is essential to establish stage-specific T1DM models to systematically evaluate the impact of T1DM onset timing on the functional integrity of the HPOU axis.

### Limitations of the study

scRNA-seq represents a robust methodology for systematically identifying distinct cellular populations within a specified tissue. Nonetheless, this technique inherently fails to preserve spatial information, which can lead to suboptimal characterization of cell types and their physiological functions. Although our single-cell atlas of the NHP HPOU axis constitutes an indispensable resource for investigating transcriptional dynamics between T1DC and T1DM conditions, it does present certain limitations. Specifically, owing to funding constraints and the considerable cost of NHP research, this study was conducted using only four cynomolgus monkeys, which may have limited its statistical power. Furthermore, although the observed transcriptional changes provide valuable insights into the potential mechanism underlying the etiology of T1DM, we fully acknowledge that further functional experiments are necessary to definitively establish the causality of these findings, thereby advancing our understanding of this condition with enhanced resolution and depth.

## Materials and methods

### Ethics statement

The use of cynomolgus monkeys (*Macaca fascicularis*) and the experimental procedures in this study were evaluated and approved by the Primate Life Sciences Ethics Committee of the Centre for Excellence in Brain Science and Intelligence Technology, Chinese Academy of Sciences (CEBSIT-2022019), in accordance with the guidelines of the Association for Assessment and Accreditation of Laboratory Animal Care. The use of mice and the experimental procedures in this study were evaluated and approved by the Institutional Animal Care and Use Committee of Guangdong Second Provincial General Hospital (2025-DW-KZ-018-01).

### Induction of diabetes

To induce type 1 diabetic monkey models, four young female cynomolgus monkeys (*Macaca fascicularis*) that were 5–6 years of age and weighed 3.1–3.4 kg were fasted overnight. The next morning, 80 mg/kg STZ was dissolved in saline and intravenously injected into the jugular vein of anesthetized cynomolgus monkeys for a period of 5 min. After STZ administration, blood glucose levels were monitored every 4 h for a 48 h period. During the first week post injection, measurements were taken twice daily. From the second week onward, blood glucose levels were subsequently assessed twice weekly. A fasting blood glucose level > 200 mg/dL in combination with a stimulated C-peptide level < 0.5 ng/mL was considered indicative of diabetes.

To generate a type 1 diabetic mouse model, female C57BL/6 mice at 10 weeks of age were randomly assigned to one of two treatment groups: the T1DC group or the T1DM group. Mice in the T1DC group received intraperitoneal injections of vehicle buffer, while those in the T1DM group were administered a single high dose of STZ (230 mg/kg body weight) to induce experimental diabetes. The animals were maintained for 10 days post injection. Mice with fasting blood glucose levels ≥ 17 mM were considered to have developed diabetes and were included in the T1DM group for subsequent analyses.

### Tissue digestion and library construction

The hypothalamus, pituitary gland, ovary, and uterus were harvested from anesthetized monkeys at six months following successful induction of diabetes and on the eighth day after the completion of menstruation, corresponding to the early follicular phase of the ovary and proliferative phase of the endometrium. Specifically, the animals were deeply anesthetized via intramuscular injection of a mixture of tiletamine hydrochloride and zolazepam hydrochloride (25 mg/kg), followed by exsanguination for euthanasia. The scalp and cranial muscles were subsequently dissected to expose the skull. Circular craniotomy was performed using a bone saw to fully expose the brain, which was then carefully removed in its entirety. The brain was then bisected sagittally along the midline to separate the left and right hemispheres and expose the ventral structures. The hypothalamus was dissected from each hemisphere on the basis of precise anatomical landmarks: bounded anteriorly by the optic chiasm, posteriorly by the posterior margin of the mammillary bodies, and laterally by the hypothalamic sulcus. The hypothalamic tissue from one hemisphere was subsequently processed for single-cell transcriptome sequencing, while the contralateral counterpart was preserved for paraffin sectioning. Concurrently, the pituitary gland was isolated by exposing the cranial base via a ventral approach after brain removal. The bony structures surrounding the sella turcica were carefully removed, and the intact pituitary gland was completely excised using fine forceps. The isolated whole pituitary tissue was subsequently processed for single-cell transcriptome sequencing.

Single-cell suspensions were subsequently prepared from four tissues using a standardized enzymatic dissociation protocol with a tumor dissociation kit (Miltenyi Biotec, #130-095-929) according to the manufacturer’s instructions. Following tissue digestion, cell viability and concentration were assessed to ensure optimal conditions for droplet-based encapsulation. The cell suspensions with a viability of > 85% and a density ranging from 300 to 600 cells per microliter were subsequently loaded onto the 10X Genomics Chromium platform for the generation of gel bead-in-emulsion (GEM) partitions. cDNA synthesis was performed within each GEM through reverse transcription, incorporating unique molecular identifiers (UMIs) for subsequent transcript quantification. After emulsion breakage, the cDNA was amplified via PCR with a total of 12 amplification cycles. Library preparation was then carried out following the Chromium Single-Cell 3′ Reagent Version 3 Chemistry protocol (10X Genomics). The fragment size distribution of the resulting libraries was evaluated using a fragment analyzer with a High-Sensitivity NGS Analysis Kit (Advanced Analytical Technologies), and the library concentration was determined by qPCR using a KAPA Library Quantification Kit for Illumina (Kapa Biosystems). Paired-end sequencing (2× 150 bp) was performed on an Illumina NovaSeq 6000 system, generating high-throughput transcriptomic profiles suitable for downstream scRNA-seq analysis.

### Processing of scRNA-seq data

Sequencing data generated on the Illumina NovaSeq 6000 platform were demultiplexed using bcl2fastq (version 2.20) to produce demultiplexed FASTQ files. A custom reference genome for *Macaca fascicularis* (version Macaca_fascicularis_6.0) was constructed in accordance with the specifications of the Cell Ranger (version 4.0.0) pipeline. The FASTQ files were subsequently processed using the count function within Cell Ranger under default parameters, which included alignment to the *Macaca fascicularis* reference genome using STAR, quality filtering, and UMI-based transcript counting. The resulting gene expression matrices were imported into R using the Read10X function from the Seurat package (version 4.1.0)^47^. Low-quality cells were filtered on the basis of predefined criteria: those expressing fewer than 200 unique genes or exhibiting a mitochondrial gene content exceeding 5% of total transcripts were excluded from further analysis. Doublets were identified and removed by the R package DoubletFinder^48^, and ambient RNA decontamination was performed using the R package DecontX^49^, both with default settings. Next, expression data were normalized using SCTransform, and PCA was conducted on the filtered and normalized dataset to reduce dimensionality. To further correct for technical variability across samples, batch effect removal was performed using the Harmony algorithm^50^. Finally, the cells were clustered using Seurat’s FindClusters function with a resolution parameter optimized for biological heterogeneity, and the number of principal components used was determined by ElbowPlot. The results were visualized in two dimensions via UMAP^51^. Cluster annotation was achieved by identifying differentially expressed marker genes using the FindAllMarkers function in Seurat with default settings and canonical markers in the literature, allowing for robust classification of cell populations on the basis of transcriptional signatures.

### Cell–cell interaction analysis

Cell–cell communication analysis was performed using the CellChat^52^ R package (v1; available at https://github.com/sqjin/CellChat). Briefly, a CellChat object was constructed on the basis of the processed scRNA-seq data. The analysis was conducted using the built-in ‘CellChatDB.human’ database as a reference for ligand–receptor interactions. Default parameters were applied to infer potential cellular communication networks and signaling strengths across cell populations.

### Pseudotime analysis

Pseudotime analysis of granulosa cells was conducted using the Monocle 2 R package to reconstruct putative developmental trajectories^53^. A gene expression count matrix was used as input to generate a new monocle object. Genes whose expression significantly differed across distinct cell clusters were selected as ordered genes, which served to infer the progression path of cellular states during differentiation. These ordered genes were subsequently employed to model lineage trajectories and infer pseudotime trajectories. To assess the reliability of the reconstructed trajectories, the DEGs were clustered and visualized according to their expression trends across the pseudotime trajectory.

### GO analysis

GO enrichment analysis was carried out using the MetaScape web-based platform (http://metascape.org/gp/index.html; version 3.5). Only GO terms with a *P* value ≤ 0.05 were considered statistically significant.

### Transcription factor analysis

To investigate the dynamic expression patterns of transcription factors (TFs) during FGC and somatic cell development, we retrieved a comprehensive list of 1419 monkey TFs from Animal TFDB 4.0 (https://guolab.wchscu.cn/AnimalTFDB4). Afterwards, the DEGs across different cell clusters and monkey TFs were intersected to obtain differentially expressed TFs, which were further visualized using a heatmap.

### DNA methylation sequencing and analysis

To assess genome-wide DNA methylation patterns, we employed WGBS. Although methylation profiling was initially intended for hypothalamic and pituitary tissues, the limited amount of tissue obtained from these organs was insufficient for library construction; therefore, only ovarian and uterine tissues were included in the present study. Genomic DNA was extracted from ovarian and uterine tissues using a commercial DNA extraction kit according to the manufacturer’s instructions. Bisulfite conversion of DNA was carried out using the Invitrogen MethylCode Bisulfite Conversion Kit following the manufacturer’s protocol. Following bisulfite treatment, the DNA was fragmented and used for WGBS library preparation. Libraries were purified using Agencourt AMPure XP beads and size-selected (200–700 bp) using a QIAquick Gel Extraction Kit (Qiagen). Libraries were sequenced on the Illumina platform to generate paired-end reads. Raw sequencing data were processed using Trim Galore to remove low-quality reads and adapter sequences, resulting in high-quality clean reads. Alignments to the *Macaca fascicularis* reference genome and methylation calling were performed using Bismark software^54^. DNA methylation levels across CpG sites were quantified and analyzed using the R package methylKit^55^.

### H&E staining

Tissue sections were prepared from the paraffin-embedded blocks by cutting 5-μm-thick sections using a rotary microtome (Leica RM2235; Leica Biosystems). Prior to staining, the slides were deparaffinized in xylene, followed by rehydration through graded ethanol solutions. The sections were subsequently washed with phosphate-buffered saline (PBS). For H&E staining, the sections were first stained with Harris hematoxylin solution (Sigma-Aldrich) followed by differentiation in 1% acid alcohol to remove excess stain and improve nuclear contrast. The sections were then briefly rinsed under running tap water for 1 min to develop the hematoxylin stain. The sections were subsequently counterstained with eosin Y solution (Sigma-Aldrich) to visualize the cytoplasmic structures. After staining, the sections were dehydrated through an ascending series of ethanol concentrations, cleared in xylene, and mounted with coverslips. Quantitative analysis was subsequently performed by counting the number of antral follicles in multiple ovarian sections, as well as the number of functionalis glands in uterine sections obtained from different regions.

### RNA isolation and qRT-PCR

Total RNA was isolated from the hypothalamus, pituitary, ovary, and uterus tissues of type 1 diabetic and control mice using a TransZol RNA Extraction Kit (TransGen, Beijing, China; ET101-01-V2) and was then reverse transcribed into cDNA using TransScript Uni All-in-One First-Strand cDNA Synthesis SuperMix (TransGen, Beijing, China; AU341). qRT-PCR was performed using PerfectStart Universal Green qPCR SuperMix (TransGen, Beijing, China; AQ631). The relative expression levels were calculated using the 2^−ΔΔCt^ method. The primers used in this study are listed below:

*Txnip*: F: TCAATACCCCTGACCTAATGGC; R: TTCTGTCAATTCGAGCAGAGAC.

*β-actin* F: TGGGTATGGAATCCTGTGGC; R: CCAGACAGCACTGTGTTGGC.

### Immunofluorescence analysis

For immunofluorescence staining, paraffin-embedded tissue sections (5-μm-thick) were deparaffinized in xylene and rehydrated through a graded ethanol series in PBS. Antigen retrieval was performed by heating the sections in citrate buffer (pH 6.0) at 98 °C for 20 min, followed by cooling to room temperature. To block nonspecific binding and permeabilize the cell membranes, the sections were incubated in blocking solution containing 5% bovine serum albumin and 0.3% Triton X-100 in PBS for 1 h at room temperature. The sections were then incubated overnight at 4 °C with primary antibodies diluted in blocking buffer. The following primary antibody was used: anti-SFRP4 (ABclonal, A4189, 1:200). Following three washes with PBS, the sections were incubated with species-appropriate fluorescently labeled secondary antibodies for 1 h at room temperature. Nuclei were counterstained with 4′,6-diamidino-2-phenylindole (DAPI; Life Technologies). Finally, the sections were mounted under coverslips and examined using a confocal laser scanning microscope.

### Biochemical assays

Blood samples were collected from *Macaca fascicularis* to assess metabolic profiles. Serum was separated from blood using a serum separator tube after allowing for the blood samples to clot for 2 h at room temperature, followed by centrifugation at 1000× *g* for 15 min. Next, the serum was immediately analyzed to determine glucose, total cholesterol, triglyceride, ALT, AST and blood urea nitrogen levels using a veterinary-specific automated chemistry analyzer (Catalyst One; IDEXX Laboratories, Inc., Westbrook, ME, USA). Specific dry-slide reagent kits were used for each analyte: glucose (Cat# 98-11076-01), cholesterol (Cat# 98-11072-01), triglyceride (Cat# 98-11086-01), alanine aminotransferase (Cat# 98-11067-01), aspartate aminotransferase (Cat# 98-11069-01) and blood urea nitrogen (Cat# 98-11070-01), ensuring high specificity and precision according to the manufacturer’s protocols. For C-peptide and FSH assessment, serum was separated as described above and stored at −80 °C until analysis. Serum C-peptide and FSH concentrations were quantified using species-specific enzyme-linked immunosorbent assay (ELISA) kits designed for monkeys (C-peptide: Cat# CSB-E13632MK; Follicle-stimulating hormone: Cat# CSB-E06866Mo). All the assays were performed in strict accordance with the manufacturer’s instructions. The data acquisition and initial analysis were conducted using SkanIt Software version 6.0.2 (Thermo Fisher Scientific). The final C-peptide and FSH concentrations were calculated on the basis of standard curves generated from known standards included in each plate.

## Supplementary information

Supplementary information

Supplementary Dataset S1

Supplementary Dataset S2

Supplementary Dataset S3

Supplementary Dataset S4

Supplementary Dataset S5

Supplementary Dataset S6

## Data Availability

The raw sequence data reported in this paper have been deposited in the Genome Sequence Archive under the accession number CRA029981 (https://ngdc.cncb.ac.cn/gsa) in National Genomics Data Center, China National Center for Bioinformation/Beijing Institute of Genomics, Chinese Academy of Sciences. The R code used for data processing is publicly available at GitHub at https://github.com/zhaozhenghui/Single-cell-atlas-of-reproductive-endocrine-organs-in-non-human-primate.
