# Supplementary material for: Single-cell atlas of reproductive endocrine organs reveals transcriptomic responses to type 1 diabetes mellitus in nonhuman primates
Source: Cell Discov. 2026 Aug 4;12:58. doi: 10.1038/s41421-026-00908-2 (PMC13439465; doi:10.1038/s41421-026-00908-2)
Supplement: Supplementary file 1 [file 41421_2026_908_MOESM1_ESM.pdf]

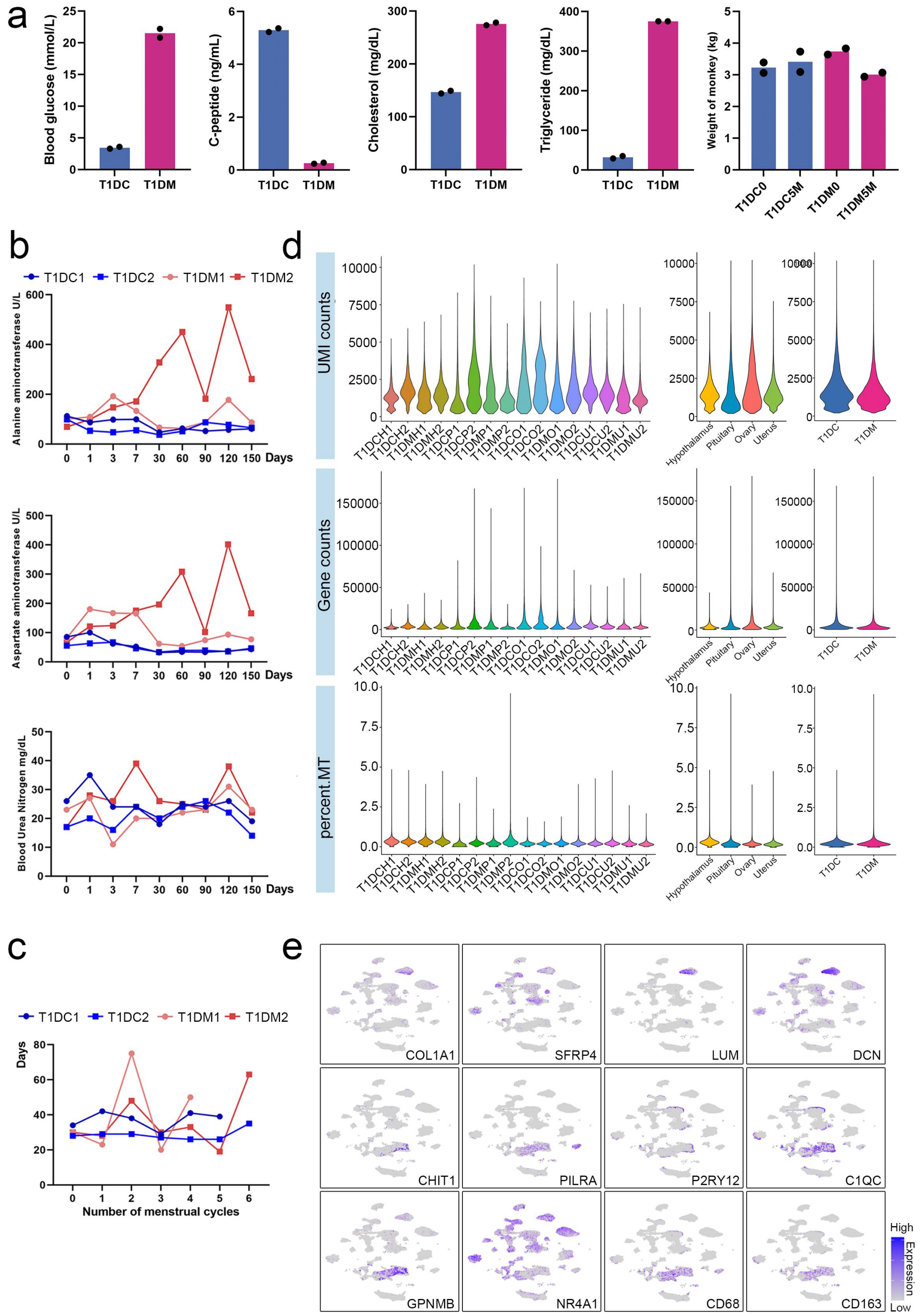

Supplementary Fig. S1. Overview of single cell transcriptional atlas. a Bar plots showing fasting blood glucose, C-peptide, Cholesterol, triglyceride, and weight in T1DC and T1DM monkeys. **b** Dynamic alterations in alanine aminotransferase (ALT), aspartate aminotransferase (AST), and blood urea nitrogen (BUN) levels in non-human primates with T1DC and T1DM. **c** Dynamics of the menstrual cycle between T1DC and T1DM monkeys. **d** Violin plot showing the UMI counts, gene counts and percentage of mitochondrial genes in each sample, organ and group. **e** Expression of selected markers identifying different clusters cast on the UMAP plot. Purple (or gray) represents a high (or low) expression level as shown on the color key at the right bottom.

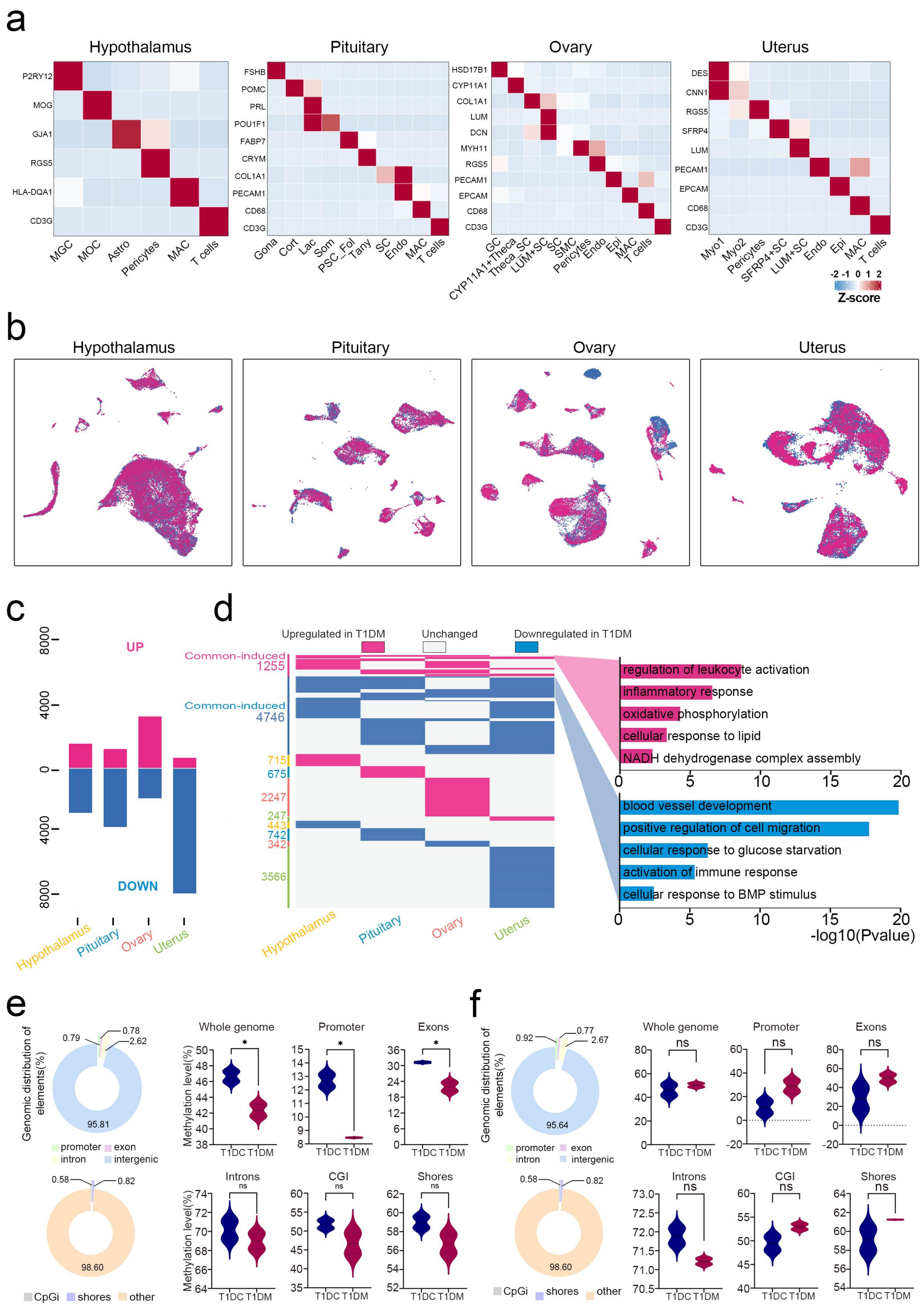

Supplementary Fig. S2. Transcriptional changes to diabetes across reproductive endocrine organs. a Heatmap showing cell type-specific marker genes. **b** UMAP plot showing the distribution of cell types. Cells are colored by T1DC (blue) and T1DM (red) groups. **c** Number of upregulated (red bar) and downregulated (blue bar) genes in the reproductive endocrine organs. **d** Distribution of differentially expressed genes in a heatmap indicating whether a gene (row) is a DEG in a given organ (column) (left). Gene ontology terms associated with top 800 common induced DEGs (right). **e** The relative proportion of differentially methylated regions (DMRs) across the genome (top) and the methylation levels for genomic features in ovary. **f** The relative proportion of DMRs across the genome (top) and the methylation levels for genomic features in uterus.

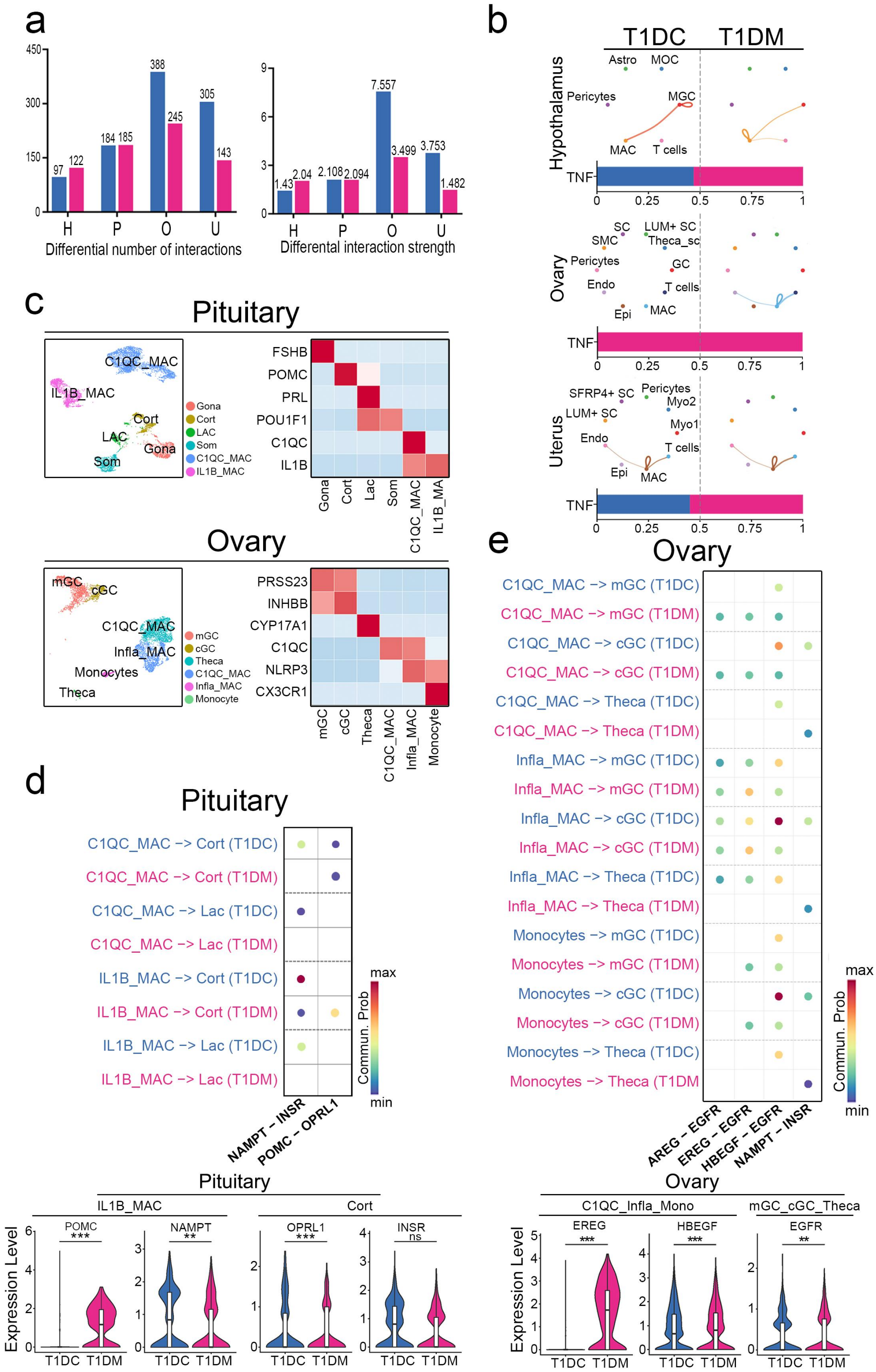

Supplementary Fig. S3. Differential signaling pathway in the pituitary and ovary. a Bar plots showing the number of inferred interactions and interaction strength in the communication network. **b** The TNF signaling pathways in T1DC (blue) and T1DM (red). **c** Subpopulations and marker genes of cell types in the pituitary and ovary. **d** The signaling pairs between macrophages and endocrine cells in pituitary (top panel); The changes of signaling pathway in the pituitary (bottom panel). **e** The signaling pairs between macrophages/monocytes and endocrine cells in ovary (top panel); The changes of signaling pathway in the ovary (bottom panel).

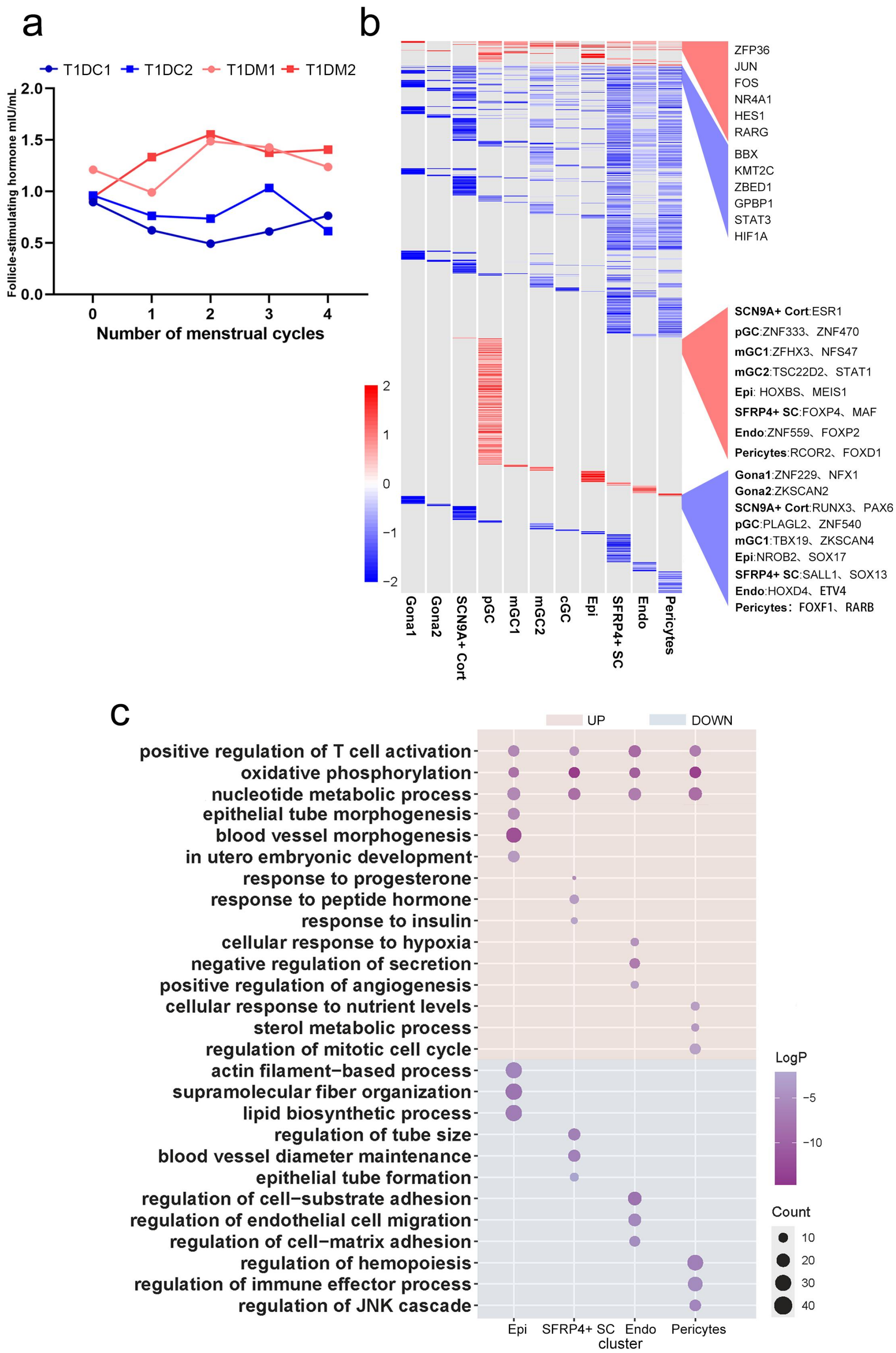

Supplementary Fig. S4. Diabetes-related transcriptional alterations in various cell types. a Dynamic alterations in Follicle-stimulating hormone (FSH) levels in non-human primates with T1DC and T1DM. **b** Heatmap showing differentially expressed transcription factors across different cell types. **c** Gene ontology terms associated with DEGs in uterine given clusters.

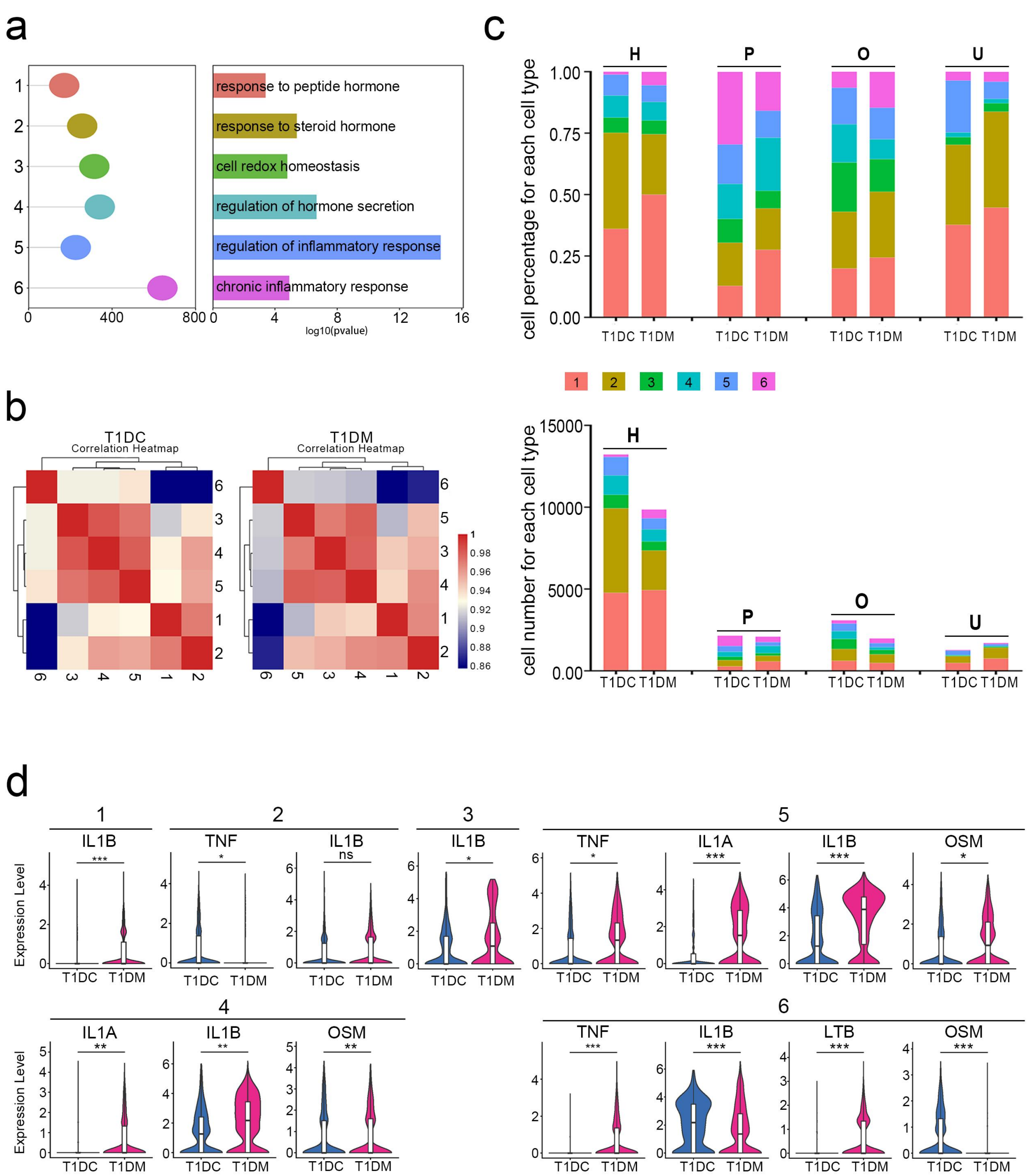

Supplementary Fig. S5. Changes of macrophages and molecular diversity of granulosa cells. a Lollipop chart illustrates the numbers of marker genes for MAC subpopulations (left panel), and bar plot represents the subtype-specific enriched biological processes. **b** Correlation analysis of macrophage subtypes between T1DC and T1DM groups. **c** The bar plot shows the relative proportions of macrophages subtypes of different groups on the top panel; The bar plot shows the numbers of macrophages subtypes of different groups at the bottom panel. **d** Violin plots showing key pro-inflammatory factors in different MAC subpopulations between T1DC and T1DM groups.

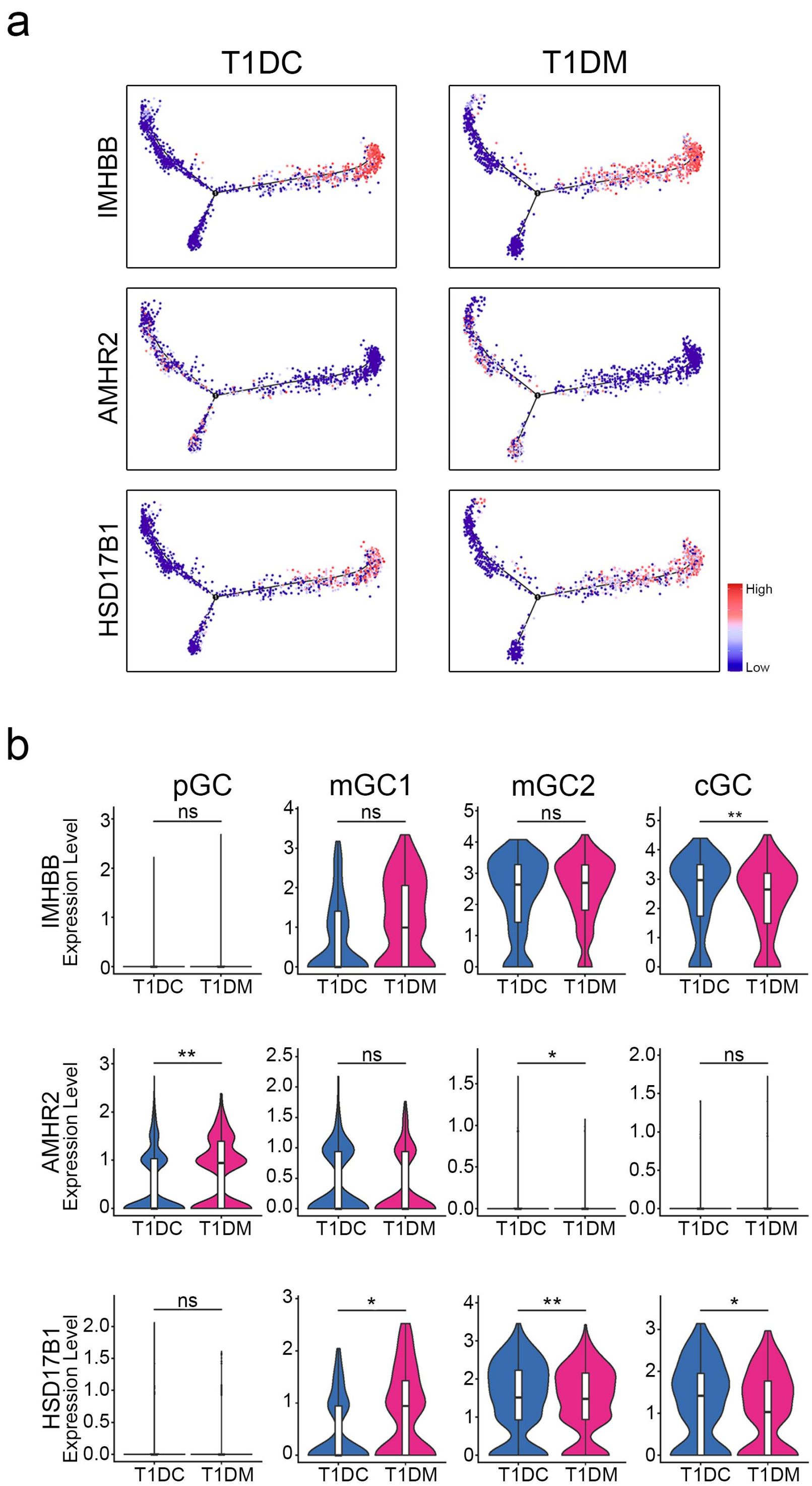

Supplementary Fig. S6. Molecular diversity of granulosa cells. a Expression of key genes along granulosa cell pseudotime trajectory. **b** Violin plots showing key genes in different granulosa cell subpopulations between T1DC and T1DM groups.

Supplementary Table S1. Sequencing information.

Hypophysis

Name	T1DC1	T1DC2	T1DM1	T1DM2
**Estimated Number of Cells**	10051	14233	8567	9314
**Mean Reads per Cell**	41786	30210	51967	47057
**Median Genes per Cell**	1716	1233	1217	1442
**Number of Reads**	419995280	429983370	445202403	438286950
**Valid Barcodes**	98.3%	97.5%	97.4%	98.3%
**Valid UMIs**	99.9%	99.9%	99.9%	99.9%
**Sequencing Saturation**	59.8%	62.1%	73.6%	68.2%
**Q30 Bases in Barcodes**	96.3%	97.1%	96.8%	95.9%
**Q30 Bases in RNA Read**	91.1%	92.7%	90.6%	90.9%
**Q30 Bases in UMI**	95.9%	96.8%	96.6%	95.8%
**Reads Mapped to Genome**	93.4%	92.4%	90.8%	93.5%
**Reads Mapped Confidently to Genome**	89.1%	87.9%	86.0%	88.3%
**Reads Mapped Confidently to Intergenic Regions**	13.0%	13.8%	13.0%	12.6%
**Reads Mapped Confidently to Intronic Regions**	40.9%	34.6%	37.4%	40.4%
**Reads Mapped Confidently to Exonic Regions**	35.2%	39.4%	35.7%	35.3%
**Reads Mapped Confidently to Transcriptome**	31.7%	34.6%	31.6%	31.6%
**Reads Mapped Antisense to Gene**	0.9%	1.6%	1.4%	0.8%
**Estimated Number of Cells**	10051	14233	8567	9314
**Fraction Reads in Cells**	86.8%	77.3%	75.4%	84.2%
**Mean Reads per Cell**	41786	30210	51967	47057
**Median Genes per Cell**	1716	1233	1217	1442
**Total Genes Detected**	17383	17371	17494	17359
**Median UMI Counts per Cell**	3706	2480	2470	3178

Pituitary

Name	T1DC1	T1DC2	T1DM1	T1DM2
**Estimated Number of Cells**	6222	11554	9158	3123
**Mean Reads per Cell**	66969	46385	49485	141814
**Median Genes per Cell**	2064	807	1279	949
**Number of Reads**	416682996	535935597	453181525	442884961
**Valid Barcodes**	98.3%	98.1%	97.7%	98.4%
**Valid UMIs**	99.9%	99.9%	99.9%	99.9%
**Sequencing Saturation**	63.4%	68.6%	67.1%	93.4%
**Q30 Bases in Barcodes**	96.3%	97.1%	96.8%	95.9%
**Q30 Bases in RNA Read**	90.7%	92.6%	91.3%	90.7%
**Q30 Bases in UMI**	95.9%	96.8%	96.7%	95.8%
**Reads Mapped to Genome**	93.4%	93.0%	91.9%	91.3%
**Reads Mapped Confidently to Genome**	89.2%	85.2%	86.7%	86.8%
**Reads Mapped Confidently to Intergenic Regions**	14.2%	9.8%	13.4%	13.3%
**Reads Mapped Confidently to Intronic Regions**	34.5%	25.0%	28.8%	31.1%
**Reads Mapped Confidently to Exonic Regions**	40.5%	50.4%	44.5%	42.4%
**Reads Mapped Confidently to Transcriptome**	36.7%	46.2%	40.3%	38.2%
**Reads Mapped Antisense to Gene**	0.7%	1.1%	1.1%	1.1%
**Estimated Number of Cells**	6222	11554	9158	3123
**Fraction Reads in Cells**	89.9%	85.5%	84.4%	74.0%
**Mean Reads per Cell**	66969	46385	49485	141814
**Median Genes per Cell**	2064	807	1279	949
**Total Genes Detected**	18460	17812	18277	16712
**Median UMI Counts per Cell**	5369	2734	3535	1801

Ovary

Name	T1DC1	T1DC2	T1DM1	T1DM2
**Estimated Number of Cells**	8898	9516	10954	8521
**Mean Reads per Cell**	47383	49948	46402	50913
**Median Genes per Cell**	2098	1679	1310	1619
**Number of Reads**	421611844	475302747	508292279	433828121
**Valid Barcodes**	98.3%	97.7%	97.7%	98.2%
**Valid UMIs**	99.9%	99.9%	99.9%	99.9%
**Sequencing Saturation**	53.2%	50.2%	68.3%	65.0%
**Q30 Bases in Barcodes**	96.2%	97.2%	96.7%	95.9%
**Q30 Bases in RNA Read**	90.9%	91.8%	90.9%	90.7%
**Q30 Bases in UMI**	95.9%	96.9%	96.6%	95.9%
**Reads Mapped to Genome**	94.7%	91.2%	91.6%	93.1%
**Reads Mapped Confidently to Genome**	90.0%	85.1%	85.2%	87.8%
**Reads Mapped Confidently to Intergenic Regions**	11.2%	13.6%	13.9%	13.8%
**Reads Mapped Confidently to Intronic Regions**	30.3%	26.9%	25.9%	27.8%
**Reads Mapped Confidently to Exonic Regions**	48.4%	44.6%	45.4%	46.2%
**Reads Mapped Confidently to Transcriptome**	44.4%	40.4%	41.3%	42.2%
**Reads Mapped Antisense to Gene**	0.7%	1.2%	1.1%	0.8%
**Estimated Number of Cells**	8898	9516	10954	8521
**Fraction Reads in Cells**	93.0%	86.5%	84.6%	89.4%
**Mean Reads per Cell**	47383	49948	46402	50913
**Median Genes per Cell**	2098	1679	1310	1619
**Total Genes Detected**	17985	18082	17930	17963
**Median UMI Counts per Cell**	5851	4895	3040	4143

Uterus

Name	T1DC1	T1DC2	T1DM1	T1DM2
Estimated Number of Cells	9106	6549	4536	10570
Mean Reads per Cell	47258	61512	101331	38529
Median Genes per Cell	1460	1696	1172	1071
Number of Reads	430327501	402839886	459638315	407248519
Valid Barcodes	98.1%	97.2%	97.1%	98.0%
Valid UMIs	99.9%	99.9%	99.9%	99.9%
Sequencing Saturation	68.4%	73.0%	86.0%	77.3%
Q30 Bases in Barcodes	96.1%	96.6%	96.8%	95.7%
Q30 Bases in RNA Read	91.3%	90.5%	91.1%	90.2%
Q30 Bases in UMI	95.9%	96.2%	96.6%	95.7%
Reads Mapped to Genome	94.0%	91.8%	91.9%	92.5%
Reads Mapped Confidently to Genome	89.1%	85.5%	85.1%	87.3%
Reads Mapped Confidently to Intergenic Regions	15.8%	14.3%	14.5%	14.4%
Reads Mapped Confidently to Intronic Regions	39.0%	28.5%	32.9%	28.6%
Reads Mapped Confidently to Exonic Regions	34.3%	42.7%	37.7%	44.2%
Reads Mapped Confidently to Transcriptome	31.1%	38.8%	34.0%	40.1%
Reads Mapped Antisense to Gene	0.8%	1.0%	1.2%	1.6%
Estimated Number of Cells	9106	6549	4536	10570
Fraction Reads in Cells	88.8%	86.4%	71.5%	89.7%
Mean Reads per Cell	47258	61512	101331	38529
Median Genes per Cell	1460	1696	1172	1071
Total Genes Detected	17738	17374	16888	17251
Median UMI Counts per Cell	3302	4270	2644	2512

Supplementary Table S2. Marker gene of cells in the literature.

	Cell Type	Marker gene	Species	Reference
	MOC	MOG	Human	[1]
	NEC	FSHB	Human	[2]
	GC	HSD17B1	Human	[3]
	Theca	STAR	Human	[4]
	SC	COL1A1	Human	[1]
	Endo	PECAM1	Human	[2]
	Epi	EPCAM	Human	[5]
	Pericytes	RGS5	Human	[1]
	SMC	MYH11	Human	[3]
	MAC	C1QC	Human	[6]
	Monocytes	CD14	Human	[7]
	T cells	CD3G	Human	[1]
	Pro cells	MKI67	Human	[8]
Hypothalamus	MGC	P2RY12	Human	[9]
	MOC	MOG	Human	[10]
	Astro	GJA1	Mouse	[11]
	Pericytes	RGS5	Human	[1]
	MAC	HLA-DQA1	Human	[1]
	T cells	CD3G	Human	[1]
Pituitary	Gona	FSHR	Human	[2]
	Cort	POMC	Human	[2]
	Lac	PRL	Human	[2]
	Som	POU1F1	Human	[2]
	PSC_Fol	FABP7	Human	[10]
	Tany	CRYM	Human	[1]
	SC	COL1A1	Human	[1]
	Endo	PECAM1	Human	[2]
	MAC	CD68	Human	[3]
	T cells	CD3G	Human	[1]
Ovary	GC	HSD17B1	Human	[3]
	CYP11A1^{+}Theca	CYP11A1	Human	[12]
	Theca_SC	COL1A1	Human	[1]
	LUM^{+}SC	LUM	Human	[3]
	SC	DCN	Human	[1]
	SMC	MYH11	Human	[3]
	Pericytes	RGS5	Human	[1]
	Endo	PECAM1	Human	[2]
	Epi	EPCAM	Human	[13]

	MAC	CD68	Human	[3]
	T cells	CD3G	Human	[1]
Uterus	Myo1	DES	Human	[13]
	Myo2	CNN1	Human	[13]
	Pericytes	RGS5	Human	[1]
	SFRP4⁺SC	SFRP4	Human	[13]
	LUM⁺SC	LUM	Human	[13]
	Endo	PECAM1	Human	[2]
	Epi	EPCAM	Human	[13]
	MAC	CD68	Human	[3]
	T cells	CD3G	Human	[1]

1. Zhou, C. *et al.* Single-Cell Atlas of Human Ovaries Reveals The Role Of The Pyroptotic Macrophage in Ovarian Aging. *Adv Sci (Weinh)* **11**, e2305175 (2024). https://doi.org/10.1002/advs.202305175
2. Zhang, S. *et al.* Single-cell transcriptomics identifies divergent developmental lineage trajectories during human pituitary development. *Nat Commun* **11**, 5275 (2020). https://doi.org/10.1038/s41467-020-19012-4
3. Fan, X. *et al.* Single-cell reconstruction of follicular remodeling in the human adult ovary. *Nat Commun* **10**, 3164 (2019). https://doi.org/10.1038/s41467-019-11036-9
4. Wang, S. *et al.* Single-Cell Transcriptomic Atlas of Primate Ovarian Aging. *Cell* **180**, 585–600.e519 (2020). https://doi.org/10.1016/j.cell.2020.01.009
5. Honardoost, M. A. *et al.* Systematic immune cell dysregulation and molecular subtypes revealed by single-cell RNA-seq of subjects with type 1 diabetes. *Genome Med* **16**, 45 (2024). https://doi.org/10.1186/s13073-024-01300-z
6. Zhang, H. *et al.* Spatially resolved C1QC+ macrophage-CD4+ T cell niche in colorectal cancer microenvironment: implications for immunotherapy response. *Cell Discovery* **11** (2025). https://doi.org/10.1038/s41421-025-00811-2
7. Hornburg, M. *et al.* Single-cell dissection of cellular components and interactions shaping the tumor immune phenotypes in ovarian cancer. *Cancer Cell* **39**, 928–944.e926 (2021). https://doi.org/10.1016/j.ccell.2021.04.004
8. Chen, M. M. *et al.* Integrated single-cell and spatial transcriptomics uncover distinct cellular subtypes involved in neural invasion in pancreatic cancer. *Cancer Cell* **43**, 1656–1676 e1610 (2025). https://doi.org/10.1016/j.ccell.2025.06.020
9. Sun, N. *et al.* Human microglial state dynamics in Alzheimer's disease progression. *Cell* **186**, 4386–4403.e4329 (2023). https://doi.org/10.1016/j.cell.2023.08.037
10. Herb, B. R. *et al.* Single-cell genomics reveals region-specific developmental trajectories underlying neuronal diversity in the human hypothalamus. *Sci Adv* **9**, eadf6251 (2023). https://doi.org/10.1126/sciadv.adf6251
11. Ren, D. *et al.* GJA1-20K Enhances Mitochondria Transfer from Astrocytes to Neurons via Cx43-TnTs After Traumatic Brain Injury. *Cellular and Molecular Neurobiology* **42**, 1887–1895 (2021). https://doi.org/10.1007/s10571-021-01070-x
12. Jones, A. S. K. *et al.* Cellular atlas of the human ovary using morphologically guided spatial transcriptomics and single-cell sequencing. *Science Advances* **10**, eadm7506 https://doi.org/10.1126/sciadv.adm7506
13. Wang, R. *et al.* Single-cell RNA sequencing identifies the prolactin receptor as a therapeutic target in adenomyosis. *Signal Transduct Target Ther* **10**, 258 (2025). https://doi.org/10.1038/s41392-025-02339-z

Supplementary Table S3.

GO terms and associated genes for Fig. 2c

Up

oxidative phosphorylation	*COX6C,COX7C,ATP6,ATP8,COX1,COX2,CYTB,ND1,ND2,ND3*
positive regulation of leukocyte activation	*RHOA,B2M,CD74,HLA-DRA,IL1B,CD46*
response to steroid hormone	*RHOA,TSPO,FOS,JUN,ND3,TXNIP,RWDD1*
positive regulation of apoptotic process	*BTG1,TSPO,FRZB,NR4A1,DNAJA1,RPL26,SFRP4,SOD1,RACK1,TXNIP*
reproductive system development	*CGA,CRIP1,SOD1,ADAMTS1,SPATA22*

Down

blood vessel development	ADM,ANXA2,APOE,COL1A1,COL1A2,COL4A1,COL4A2,TYMP,FGFR1
negative regulation of cell migration	APOE,ARHGDIB,CALR,CD63,CDH11,GADD45A,DUSP1,HMOX1,IDH2
regulation of proteolysis	ANXA2,APOE,APP,CLU,PLK3,CSNK1A1,CSNK1D,CSNK1E,CSTB,DAB2
response to endoplasmic reticulum stress	ATF4,ATP2A2,CCND1,CALR,DDX3X,ERN1,PDIA3,HSPA5,MAN1A1,MAP3K5
regulation of endothelial cell proliferation	APOE,F3,FGFR1,HIF1A,HMOX1,MEF2C,SPARC,STAT1,STAT3,THBS1

GO terms and associated genes for Supplementary Fig. S2d

Up

regulation of leukocyte activation	CD2,CD6,CD80,FGR,GATA3,HLA-DOA,IGF2,IL1A,IL1B,IL2RA
inflammatory response	CD5L,CD6,CCR5,FPR2,ACKR1,GATA3,GZMA,GZMB,IL1A,IL1B
oxidative phosphorylation	COX1,ND1,ND2,ND3,ND4L,NDUFB4,NDUFS5
cellular response to lipid	CD6,CD80,CCR5,FCAR,GFI1,IL1A,IL1B,ND3,RXRG,CLDN1
NADH dehydrogenase complex assembly	ND1,ND2,NDUFS5

Down

blood vessel development	ADM,ANXA2,RHOB,BAK1,BMPR2,CDH2,CDH13,COL1A1,SLC31A1,CCN2
positive regulation of cell migration	AIF1,ANXA3,APC,RHOB,BMP2,BMPR2,C5AR1,CALR,SCARB1,CDH13
cellular response to glucose starvation	PLIN2,ASNS,CHKA,HSPA5,PIK3C3,SLC2A1,UPP1,SLC7A5,EIF2AK3
activation of immune response	BTK,C2,C5,C5AR1,CTSS,CD55,EP300,FYB1,FYN,GRB2
cellular response to BMP stimulus	BMP2,BMPR2,GDF1,SMAD4,SMAD9,PPARG,SOX9,USP15,FST,LEF1

Supplementary Table S4. Ovarian and uterine ultrasound measurements in the Type 1 diabetes model at 5 months (unit: mm).

Group	Uterus	Endometrium	Left ovary	Right ovary	Menstrual cycle
T1DC2	15.6*10.9	2.2	7.2*4.8	6.8*5.0	Cycle Day 8
T1DM1	14.0*8.5	1.9	5.8*3.8	5.6*3.3	Cycle Day 8
T1DC1	34.3*16.7	4.1	6.0*4.8	7.1*5.2	Cycle Day 22
T1DM2	15.5*8.2	2.6	4.4*3.1	4.5*3.2	Cycle Day 25
